# Synthesis of novel organophosphorus compounds via reaction of substituted 2-oxoindoline-3-ylidene with acetylenic diesters and triphenylphosphine or triphenyl phosphite

**DOI:** 10.1038/s41598-024-56774-z

**Published:** 2024-03-15

**Authors:** Mahsa Najafi, Ghasem Marandi

**Affiliations:** Department of Organic Chemistry, Faculty of Chemistry, Urmia University, Urmia, Iran

**Keywords:** 2-oxoindoline-3-ylidenes, Phosphorus ylide, Phosphonate ester, Acetylenic esters, Organic chemistry, Chemical synthesis

## Abstract

An efficient reaction between triphenylphosphine or triphenyl phosphite and 2-oxoindoline-3-ylidene derivatives in the presence of acetylenic esters leads to functionalized 2-oxoindoline-3-ylidene containing phosphorus ylieds or phosphonate esters. All compounds obtained in these reactions are stable and have good yields.

## Introduction

The synthesis and development of organic compounds, as well as the study of their reaction aspects, are interesting topics for organic chemists^1^. Phosphorus compounds have influenced many branches of science, such as chemistry, medicine, materials science, and agriculture, due to their wide range of applications^2^. Hence, the generation of this class of compounds has attracted the attention of researchers^3,4^. In the chemistry literature, each compound containing a C−P bond is organophosphorus, such as phosphorus ylides, phosphonates, phosphinates, phosphines, phosphinoxides and iminophosphorane^5^. Since 1990, many reports about the synthesis of phosphorus ylides have been published, which indicate that these compounds are important structures in various scientific fields such as chemistry, agriculture, and medicine^6–10^. In addition to the above-mentioned issues, from a scientific point of view, the special position of heterocyclic compounds, such as isatin and its derivatives, is well known to scientists in biology and industry.^11–19^.

**Figure 1:**
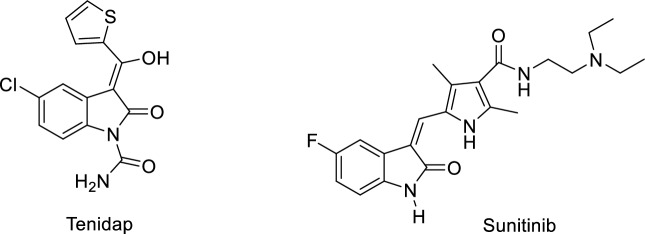
Tenidap and Sunitinib, two structure with 2-oxoindoline-3-ylidene core as anti-inflammatory agents.

The distinct heterocyclic structures, such as isatin, with their high transformation potential to other synthetic compounds, can play a key role in the synthesis of complex organic structures^20–22^. Also, these derivatives possess many biological activities such as anti-cancer^11^, anti-inflammatory^23–25^, anti-HIV^12^, anticonvulsant^26^, antibacterial^13^, antifungal^14^, anti-Parkinsonian^15^ and antiglaucomic^16^ (Fig. 1). Herein, according to our investigations^27–30^, due to the importance of organophosphorus compounds and isatin cores, we describe the synthesis of functionalized 2-oxoindoline-3-ylidene containing novel organophosphorus compounds. Therefore, we have performed a facile one-pot reaction between 2-oxoindoline-3-ylidene derivatives and triphenyphosphine or triphenyl phosphite in the presence of acetylenic esters.

## Results and discussion

**Figure 2:**
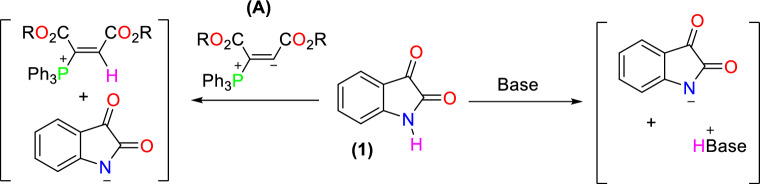
The acidic performance of NH in isatin structure.

The literature survey indicates that N–H of isatin **1** can be deprotonated in the presence of a base such as tetrabutylammonium hydroxide^31–33^. Additionally, it reacts with the vinyl phosphonium zwitterion (**A**) from the reaction between triphenylphosphine and acetylenic diesters^34^ (Fig. 2).

The reaction of isatin with active CH acid compounds via a Knoevenagel condensation reaction leads to an *α,β* unsaturated compounds^35–38^. These target structures can serve as important reagents for the synthesis of new organophosphorus compounds with potent biological activities.

**Figure 3:**
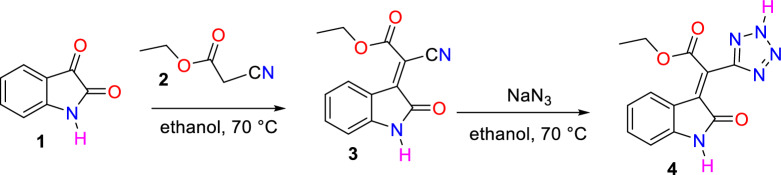
Synthesis of tetrazole-substituted 2-oxoindoline-3-ylidene **4**.

For this purpose, in the first step, isatin **1** reacts with ethyl cyanoacetate **2** in a Knoevenagel condensation to form ethyl 2-cyano-2-(2-oxoindolin-3-ylidene)acetate **3**. Then, compound **3** reacts with sodium azide in ethanol at 70 °C to obtain ethyl 2-(2-oxoindolin-3-ylidene)-2-(2*H*-tetrazol-5-yl)acetate **4** following the previous procedure (Fig. 3)^30^.

**Figure 4:**
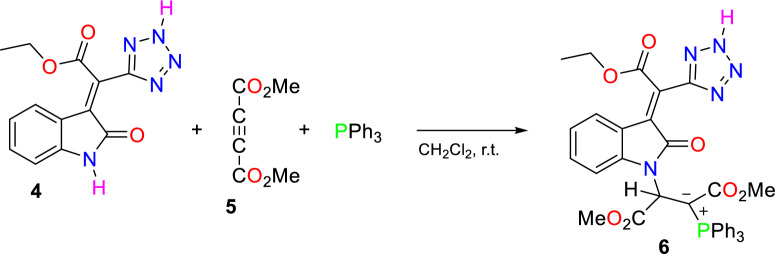
Synthesis of phosphorus ylide **6**.

At the other step, the corresponding 2-oxoindoline-3-ylidene **4** in the presence of triphenylphosphine reacts with dimethyl aceylenedicarboxylate **5** to produce phosphorus ylide **6** (Fig. 4).

Based on the well-established chemistry of trivalent phosphorus nucleophiles^2,5–9,39,40^, it is reasonable to assume that phosphorus ylide **6** results from the initial addition of triphenylphosphine to the dimethyl acetylenedicarboxylate **5** and, followed by protonation by the 1:1 adduct by the NH of 2-oxoindoline-3-ylidene **4** resulting in the formation of phosphorus ylide **6** (see Figs. 2 and 4).

**Figure 5:**
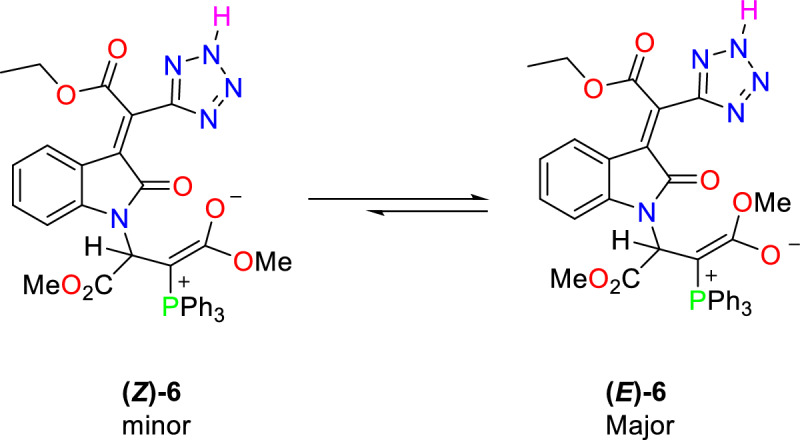
Structure of phosphorus ylide **6** as Major and minor geometrical isomers.

The ylide moiety in these compounds is highly conjugated with the adjacent carbonyl group, and rotation around the partial double bond of the (*E*)-**6** and (*Z*)-**6** geometric isomers is slow on the NMR timescale at room temperature (see Fig. 5).

**Figure 6:**
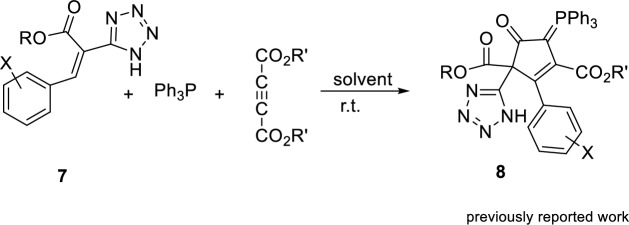
Reaction for the synthesis of tetrazole-containing cyclopentenyl phosphanylidene dicarboxylates **8**.

The structure of organophosphorus ylide **6**, indicates that the reaction between compound **4** and dimethyl acetylenedicarboxylate **5** in the presence of triphenylphosphine has occurred in a chemo-selective manner. In our previous study, we observed that the vinyl phosphonium zwitterionic intermediate (**A**) reacted with the conjugated C–C double bond instead of the NH of tetrazole, and the reaction proceeded via a Michael addition to produce the final product **8** (Fig. 6)^30^. However, in the current study, neither the, NH of tetrazole nor the conjugated C–C double bond have any reaction with the vinyl phosphonium zwitterion. Instead of reacting with them, the NH of the isatin moiety reacts with the phosphonium zwitterionic intermediate to generate product **6** in a chemo-selective manner.

The stable structure of phosphorus ylide **6** was deduced from IR, mass, ^1^H, ^13^C and ^31^P NMR spectroscopic data. The IR spectrum of compound **6** showed distinct peaks for the tetrazole N–H and carbonyl groups at 3448 and 1735 cm^-1^, respectively. The ^1^H NMR spectra of compound **6** showed four resonances for the methyl groups at δ = 3.11 and 3.77 ppm for the major rotamer and at δ = 3.77 and 4.42 ppm for the minor rotamer, respectively. Furthermore, in accordance with the major and minor structures of compound **6**, ^31^P NMR spectrum shows resonances at δ = 22.43 and 22.78 ppm.

**Figure 7:**
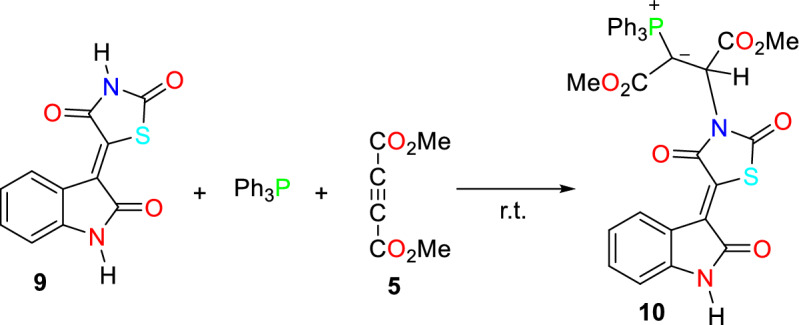
The chemo-selective synthesis of thiazolidine-2,4-dione containing phosphorus ylide **10**.

In continuation of the present work, another chemo-selective reaction occurred when compound **9** was used to generate organophosphorus compound **8** (Fig. 7).

The chemo-selectivity between the NH group of isatin and the thiazolidine-2,4-dione moieties has been determined by comparing their respective ^1^H NMR spectra. In the ^1^H NMR spectrum of compound **10**, the peak at δ = 9.76 ppm remained unchanged. In addition, the peak at 9.76 ppm was removed and appeared at 4.73 ppm in the D_2_O exchange experiment. Table 1 shows the stable structures of compounds resulting from the reaction between the NH source compound and the phosphonium zwitterionic intermediate.

**Table 1:** List of synthesized organophosphorus compounds.

Base compound	CH acid source	NH acid	R	Conditions	Product	Yield (%)
			Me	Ethyl acetate, r.t		78
			Me	Ethyl acetate, r.t		75
			Me	Di ethyl ether, r.t		86
			Et	Acetone, r.t		80
			Me	Di ethyl ether, r.t		86
			Me	Ethyl acetate, r.t		76
			Me	Ethyl acetate, r.t		88

**Figure 8:**
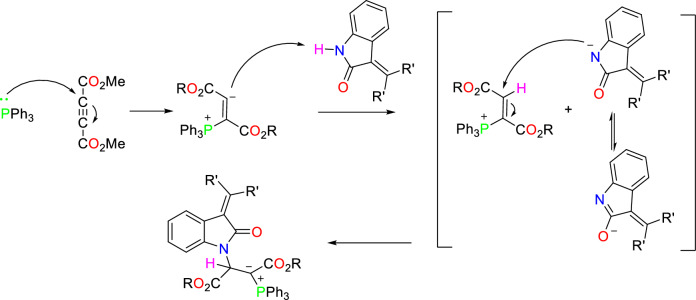
The proposed mechanism for synthesis of the phosphorus ylides.

An illustrative mechanism for the synthesis of phosphorus ylides has been shown in Fig. 8.

**Figure 9:**
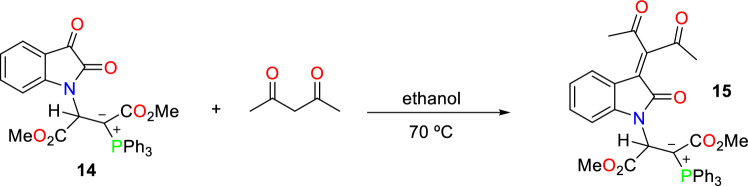
A Knovenagel condensation reaction between acetyl acetone and phosphorus ylide **14**.

In continuation of our investigations into phosphorus compounds, we have conducted another reaction between isatin derivatives and dimethyl acetylenedicarboxylate in the presence of triphenyphosphine. Then, the obtained product **14** reacts with acetyl acetone in ethanol as a solvent at 70 °C to generate phosphorus ylide **15** (Fig. 9).

**Figure 10:**
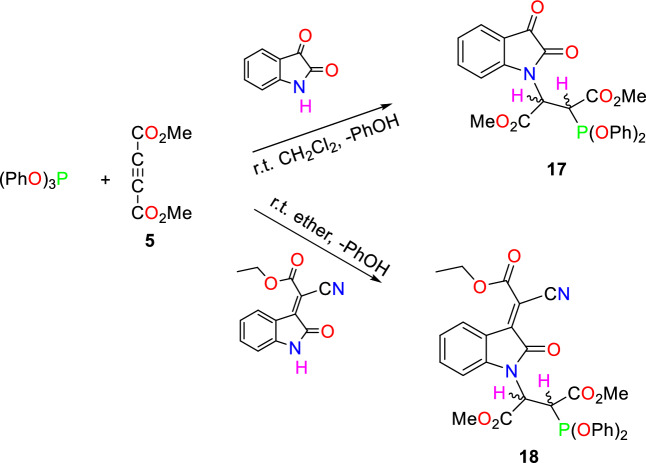
The synthesis of an isatin core containing phosphonate esters.

Another reaction was performed to synthesize isatin core containing structures by reacting triphenyl phosphite with isatin and its derivatives in the presence of dimethyl acetylenedicarboxylate **5** (Fig. 10).

According to our expectations for the synthesis of the phosphonate ester distereoisomers in this reaction, only one product was generated for each reaction. As seen in previous works, the coupling constant between Hydrogen atoms and a phosphorus atom enables us to identify the *R* or *S* configuration of chiral carbons. However, in the synthesized compounds **17** and **18** signals for these hydrogens are significantly broadened, making the measurement of ^2^*J*_PH_ and ^3^*J*_PH_ impossible^41^.

## Experimental

All melting points were measured using a Barnstead Electrothermal 9200 apparatus. In addition, the IR spectra of the synthesized compounds were recorded with a Thermo-Nicolet Nexus 670 FT-IR spectrometer. The ^1^H, ^13^C and ^31^P NMR spectra for the obtained compounds were recorded using a BRUKER DRX-250 AVANCE instruments with CDCl_3_ as the solvent and TMS as the internal standard at frequencies of (250.1, 62.9 and 101.3) MHz, respectively. The mass spectra of newly synthesized compounds were analyzed using an Agilent 5975C mass spectrometer operating at an ionization potential of 70 eV. Elemental analyses (C, H, N) were conducted using a Heraeus CHN-O-Rapid analyzer. Triphenylphosphine, triphenylphosphite, acetylenic esters, ethylcyanoacetate, sodium azide, isatin, thiazolidine-2,4-dione, malononitrile, and acetyl acetone as well as all solvents were purchased from Merck, Fluka and Sigma-Aldrich companies and used without additional purification.

### General procedure for the synthesis of NH source compounds (exemplified by 4)

To a magnetically stirred solution of ethyl cyanoacetate (0.113 g, 1 mmol) and isatin (0.147 g, 1 mmol) in EtOH (10 mL) was prepared, and then a mixture of sodium azide (0.07 g, 1.1 mmol) in EtOH (5 mL) was added dropwise over 5 min at room temperature. Then, the mixture was heated to 70 °C for 10 h to complete the reaction, which was monitored by TLC). The solvent was removed through slow evaporation. All residues were washed with cold diethyl ether (2 × 3 mL), and the desired product was then filtered and recrystallized from ethanol (3 mL).

#### Ethyl 2-(2-oxoindolin-3-ylidene)-2-(2*H*-tetrazol-5-yl)acetate (*4*)

Brown powder, Yield (0.23 g, 81%), mp: 126–128 °C; IR (KBr, υ_max_): 3439 (NH_tet_), 3370 (NH_isat_), 1718 (C=O) cm^-1^; ^1^H NMR (250 MHz, CDCl_3_): δ 1.44 (3H, t, *J* = 7 Hz, OCH_2_C*H*_3_), 4.46 (2H, q, *J* = 7 Hz, OC*H*_2_CH_3_), 6.89 (1H, d, *J* = 8 Hz, Ar*H*), 7.04 (1H, t, *J* = 7.5 Hz, Ar*H*), 7.43 (1H, t, *J* = 8.0 Hz, Ar*H*), 7.80 (1H, brs, N*H*), 8.32 (1H, d, *J* = 8.0 Hz, Ar*H*). ^13^C NMR (63.0 MHz, CDCl_3_): δ 13.9 (OCH_2_*C*H_3_), 63.4 (O*C*H_2_CH_3_), 111.0 (*C*H_Ar_), 123.2 (*C*_Ar_), 124.0 (*C* = CCO), 125.7 (*C*H_Ar_), 130.1 (*C*H_Ar_), 135.9 (*C*H_Ar_), 138.7 (C = *C*CO), 144.1 (HN*C*_Ar_), 145.0 (C_tet_), 150.0 (HN*C*O), 166.5 (*C*O_2_Et).

### General procedure for the synthesis of phosphorus ylides (exemplified by 6)

To a magnetically stirred solution of ethyl 2-(2-oxoindolin-3-ylidene)-2-(2*H*-tetrazol-5-yl)acetate **4** (0.285 g, 1 mmol) and triphenylphosphine (0.262 g, 1 mmol) in ethyl acetate (10 mL), dimethyl acetylenedicarboxylate (0.142 g, 1 m mol) in ethyl acetate (3 mL) was added dropwise at room temperature. After approximately 24 h of stirring at room temperature, the crude products were collected and washed with cold diethyl ether (2 × 3 mL).

#### *Dimethyl 2-(3-(2-ethoxy-2-oxo-1-(2H-tetrazol-5-yl)ethylidene)-2-oxoindolin-1-yl)-3-(triphenyl-λ*^*5*^*-phosphanylidene)succinate (6)*

Red powder, Yield (0.54 g, 78%), mp: 78–81 °C; IR (KBr, υ_max_): 3448 (NH_tet_), 1735 (C=O) cm^-1^; MS (*m/z*, %): 689.6 (M^+^, 1), 557.5 (4), 427.4 (1), 277.2 (100), 262.3 (49), 183.1 (58), 77.1 (62). Anal. Calcd for C_37_H_32_N_5_O_7_P (689.7): C, 64.44; H, 4.68; N, 10.15%. Found: C, 64.61; H, 4.52; N, 10.27%. Major isomer: ^1^H NMR (250 MHz, CDCl_3_): δ 1.40 (3H, brs, OCH_2_C*H*_3_), 3.11 (3H, s, OC*H*_3_), 3.77 (3H, s, OC*H*_3_), 4.33 (2H, brs, OC*H*_2_CH_3_), 5.34 (1H, d, ^3^*J*_PH_ = 19.5 Hz, C*H*), 6.84–7.10 (3H, m, Ar*H*), 7.45–7.80 (15H, m, 3 C_6_H_5_), 8.58–8.18 (1H, brs, ArH); ^13^C NMR (63.0 MHz, CDCl_3_): δ 10.8 (OCH_2_*C*H_3_), 37.3 (d, ^1^*J*_PC_ = 120.3 Hz, P = *C*), 49.1 and 49.6 (2s, 2 O*C*H_3_), 51.5 (P = C–*C*H, d, ^2^*J*_PC_ = 15.1 Hz), 60.0 (O*C*H_2_CH_3_), 125.4 (d, ^3^*J*_PC_ = 12.0 Hz, C_meta_), 126.0 (d, ^1^*J*_PC_ = 82.0 Hz, C_ipso_), 128.9 (d, ^2^*J*_PC_ = 7.0 Hz, C_ortho_), 132.6 (C_para_), 108.0 (*C*H_Ar_), 109.6 (*C*_Ar_), 125.7 (*C*H_Ar_), 130.2 (*C*H_Ar_), 131.4 (*C* = CCO_2_), 132.6 (C = *C*CO_2_), 135.2 (*C*H_Ar_), 147.4 (*C*_Ar_), 158.8 (N*C*O), 156.3 (*C*_tet_), 162.3 (*C*O_2_Et), 166.8 (d, ^3^*J*_PC_ = 11.5 Hz, *C*OCH_3_), 167.7 (d, ^2^*J*_PC_ = 14.7 Hz, P = C–*C*O); ^31^P NMR (101.2 MHz, CDCl_3_): δ 22.43 (Ph_3_P^+^–C); Minor isomer: ^1^H NMR (250 MHz, CDCl_3_): δ 1.28 (3H, brs, OCH_2_C*H*_3_), 3.77 (3H, s, OC*H*3), 4.23 (2H, brs, OC*H*_2_CH_3_), 4.42 (3H, s, OC*H*_3_), 5.23 (1H, d, ^3^*J*_PH_ = 17.8 Hz, C*H*), 6.84–7.10 (3H, m, Ar*H*), 7.45–7.80 (15H, m, 3 C_6_H_5_), 8.58–8.18 (1H, brs, ArH). ^13^C NMR (63.0 MHz, CDCl_3_) δ/ppm: 10.5 (OCH_2_*C*H_3_), 36.0 (d, ^1^*J*_PC_ = 122.1 Hz, P = *C*), 48.9 and 49.4 (2s, 2 O*C*H_3_), 47.5 (P = C–*C*H, d, ^2^*J*_PC_ = 22.1 Hz), 60.0 (O*C*H_2_CH_3_), 125.8 (d, ^3^*J*_PC_ = 12.0 Hz, C_meta_), 127.4 (d, ^1^*J*_PC_ = 104.0 Hz, C_ipso_), 128.9 (d, ^2^*J*_PC_ = 7.0 Hz, C_ortho_), 130.2 (C_para_), 110.8 (*C*H_Ar_), 111.0 (*C*_Ar_), 121.7 (*C*HAr), 130.8 (*C* = CCO), 128.1 (*C*H_Ar_), 132.6 (*C*H_Ar_), 131.0 (C = *C*CO), 146.7 (*C*_Ar_), 159.0 (N*C*O), 158.5 (*C*_tet_), 164.5 (*C*O_2_Et), 166.4 (d, ^3^*J*_PC_ = 12.3 Hz, *C*OCH_3_), 167.5 (d, ^2^*J*_PC_ = 13.8 Hz, P = C–*C*O); ^31^P NMR (101.2 MHz, CDCl_3_) δ/ppm: 22.78 (Ph_3_P^+^–C).

#### *Dimethyl 2-[2,4-dioxo-5-(2-oxoindolin-3-ylidene)thiazolidin-3-yl]-3-(triphenyl-λ*^*5*^*-phosphanylidene)succinate (10)*

Orange powder, Yield (0.49 g, 75%), mp: 80–83 °C; IR (KBr, υ_max_): 3420 (NH), 1738 (C=O) cm^-1^; MS (*m/z*, %): 650.6 (M^+^, 1), 557.6 (11), 388.3 (4), 277.3 (100), 246.3 (17), 262.3 (37), 183.1 (28), 77.1 (32). Anal. Calcd for C_35_H_27_N_2_O_7_PS (650.6): C, 64.61; H, 4.18; N, 4.31%. Found: C, 64.77; H, 4.13; N, 4.36%. Major isomer: ^1^H NMR (250 MHz, CDCl_3_): δ 3.11 (3H, s, OC*H*_3_), 3.79 (3H, s, OC*H*_3_), 5.31 (1H, d, ^3^*J*_PH_ = 16.0 Hz, C*H*), 6.85–7.82 (4H, m, Ar*H*), 7.45–7.82 (15H, m, 3 C_6_H_5_), 9.76 (1H, brs, N*H*); ^13^C NMR (63.0 MHz, CDCl_3_): δ 33.2 (d, ^1^*J*_PC_ = 100.0 Hz, P = *C*), 46.4 and 49.1 (2s, 2 O*C*H_3_), 51.5 (P = C–*C*H, d, ^2^*J*_PC_ = 15.1 Hz), 125.4 (d, ^3^*J*_PC_ = 11.3 Hz, C_meta_), 127.3 (d, ^1^*J*_PC_ = 91.4 Hz, C_ipso_), 128.9 (C_ortho_), 130.2 (C_para_), 109.6 (*C*H_Ar_), 111.2 (*C*_Ar_), 120.1 (*C*H_Ar_), 122.2 (*C*H_Ar_), 129.3 (C = *C*SCO), 130.2 (*C* = CSCO), 135.3 (*C*H_Ar_), 147.2 (*C*_Ar_), 156.3 (HN*C*O), 166.3 (d, ^3^*J*_PC_ = 11.2 Hz, *C*OCH_3_), 167.5 (d, ^2^*J*_PC_ = 13.8 Hz, P = C–*C*O), 173.0 (*C*ONCOS), 180.9 (CON*C*OS); ^31^P NMR (101.2 MHz, CDCl_3_): δ 19.45 (Ph_3_P^+^–C); Minor isomer: ^1^H NMR (250 MHz, CDCl_3_) δ/ppm: 3.70 (3H, s, OC*H*_3_), 3.79 (3H, s, OC*H*_3_), 5.23 (1H, d, ^3^*J*_PH_ = 16.0 Hz, C*H*), 6.85–7.82 (4H, m, Ar*H*), 7.45–7.82 (15H, m, 3 C_6_H_5_), 9.76 (1H, brs, N*H*); ^13^C NMR (63.0 MHz, CDCl_3_): δ 36.3 (d, ^1^*J*_PC_ = 107.9 Hz, P = *C*), 49.1 and 49.7 (2s, 2 O*C*H_3_), 52.6 (P = C–*C*H, d, ^2^*J*_PC_ = 15.0 Hz), 125.9 (d, ^3^*J*_PC_ = 11.3 Hz, C_meta_), 127.3 (d, ^1^*J*_PC_ = 91.4 Hz, C_ipso_), 129.3 (C_ortho_), 130.2 (C_para_), 107.8 (C_Ar_), 114.7 (*C*H_Ar_), 121.4 (*C*H_Ar_), 123.0 (*C*H_Ar_), 129.5 (C = *C*SCO), 130.2 (*C* = CSCO), 138.5 (*C*H_Ar_), 147.2 (*C*_Ar_), 153.6 (HN*C*O), 166.7 (d, ^2^*J*_PC_ = 13.0 Hz, P = C–*C*O), 169.8 (d, ^3^*J*_PC_ = 11.5 Hz, COCH3), 173.0 (*C*ONCOS), 181.2 (CON*C*OS); ^31^P NMR (101.2 MHz, CDCl_3_): δ 19.63 (Ph_3_P^+^–C).

#### *Dimethyl 2-[3-(1-cyano-2-ethoxy-2-oxoethylidene)-2-oxoindolin-1-yl]-4-(methylperoxy)-3-(triphenyl-λ*^*5*^*-phosphanylidene)butanoate (11)*

Dark red powder; Yield (0.56 g, 86%), mp: 82–85 °C; IR (KBr, υ_max_): 2200 (C≡N), 1750 and 1720 (C=O) cm^-1^; MS (*m/z*, %): 646.5 (M^+^, 1), 384.5 (1), 355.4 (2), 277.3 (100), 262.3 (8), 185.2 (47), 77.2 (65). Anal. Calcd for C_37_H_31_N_2_O_7_P (646.6): C, 68.73; H, 4.83; N, 4.33%. Found: C, 68.80; H, 4.72; N, 4.41%. Major isomer: ^1^H NMR (250 MHz, CDCl_3_): δ 1.43 (3H, t, ^3^*J*_HH_ = 6.8 Hz, OCH_2_C*H*_3_), 3.12 (3H, s, OC*H*_3_), 3.77 (3H, s, OC*H*_3_), 4.38 (2H, q, ^3^*J*_HH_ = 6.8 Hz, OC*H*_2_CH_3_), 5.37 (1H, d, ^3^*J*_PH_ = 16.3 Hz, C*H*), 7.10 (1H, t, ^3^*J* = 7.0 Hz, Ar*H*), 7.35–7.73 (15H, m, 3 C_6_H_5_), 7.80 (1H, brs, Ar*H*), 8.00 (1H, brs, Ar*H*), 8.18 (1H, d, ^3^*J* = 7.5 Hz, Ar*H*); ^13^C NMR (63.0 MHz, CDCl_3_): δ 14.0 (OCH_2_*C*H_3_), 29.5 (d, ^1^*J*_PC_ = 122.1 Hz, P = *C*), 52.3 and 54.4 (2s, 2 O*C*H_3_), 54.5 (P = C–*C*H, d, ^2^*J*_PC_ = 13.5 Hz), 63.3 (O*C*H_2_CH_3_), 124.5 (d, ^1^*J*_PC_ = 120.3 Hz, C_ipso_), 128.5 (d, ^3^*J*_PC_ = 12.0 Hz, C_meta_), 132.0 (C_ortho_, C_para_), 111.0 (*C*H_Ar_), 114.1 (*C*_Ar_), 125.5 (*C*H_Ar_), 130.0 (*C*H_Ar_), 133.4 (*C* = CCO_2_), 135.8 (C = *C*CO_2_), 134.6 (*C*H_Ar_), 145.5 (*C*_Ar_), 152.5 (N*C*O), 166.3 (*C*O_2_Et), 163.5 (d, ^3^*J*_PC_ = 12.0 Hz, *C*OCH_3_), 168.0 (d, ^2^*J*_PC_ = 13.7 Hz, P = C–*C*O); ^31^P NMR (101.2 MHz, CDCl_3_): δ 22.42 (Ph_3_P^+^–C); Minor isomer: ^1^H NMR (250 MHz, CDCl_3_): δ 1.23 (3H, uneven t, OCH_2_C*H*_3_), 3.49 (3H, s, OC*H*_3_), 3.68 (3H, s, OC*H*_3_), 4.30 (2H, m, OC*H*_2_CH_3_), 5.37 (1H, d, ^3^*J*_PH_ = 16.3 Hz, C*H*), 7.10 (1H, t, ^3^*J* = 7.0 Hz, Ar*H*), 7.35–7.73 (15H, m, 3 C_6_H_5_), 7.80 (1H, brs, Ar*H*), 8.00 (1H, brs, Ar*H*), 8.18 (1H, d, ^3^*J* = 7.5 Hz, Ar*H*); ^13^C NMR (63.0 MHz, CDCl_3_): δ 13.3 (OCH_2_*C*H_3_), 32.7 (d, ^1^*J*_PC_ = 119.5 Hz, P = *C*), 51.0 and 53.2 (2s, 2 O*C*H_3_), 54.5 (P = C–*C*H, d, ^2^*J*_PC_ = 13.5 Hz), 64.6 (O*C*H_2_CH_3_), 124.5 (d, ^1^*J*_PC_ = 120.3 Hz, C_ipso_), 128.9 (d, ^3^*J*_PC_ = 11.3 Hz, C_meta_), 132.3 (C_ortho_, C_para_), 112.6 (*C*H_Ar_), 114.3 (*C*_Ar_), 125.5 (*C*H_Ar_), 130.0 (*C*H_Ar_), 133.4 (*C* = CCO_2_), 135.8 (C = *C*CO_2_), 134.6 (*C*H_Ar_), 145.5 (*C*_Ar_), 153.1 (N*C*O), 166.3 (*C*O_2_Et), 164.6 (d, ^3^*J*_PC_ = 12.0 Hz, *C*OCH_3_), 170.1 (d, ^2^*J*_PC_ = 13.7 Hz, P = C–*C*O); ^31^P NMR (101.2 MHz, CDCl_3_): δ 22.75 (Ph_3_P^+^–C).

#### *Diethyl 2-(3-(1-cyano-2-ethoxy-2-oxoethylidene)-2-oxoindolin-1-yl)-3-(triphenyl-λ*^*5*^*-phosphanylidene)succinate (12)*

Dark red powder; Yield (54 g, 80%), mp: 94–97 °C; IR (KBr, υ_max_): 2230 (C≡N), 1735 (C=O) cm^-1^; MS (*m/z*, %): 674.8 (M^+^, 1), 647.5 (1), 563.1 (1), 412.4 (10), 277.3 (86), 262.3 (100), 183.2 (98), 77.1 (43). Anal. Calcd for C_39_H_37_N_2_O_7_P (676.7): C, 69.22; H, 5.51; N, 4.14%. Found: C, 69.31; H, 5.47; N, 4.22%. Major isomer: ^1^H NMR (250 MHz, CDCl_3_): δ 0.44 (3H, br s, OCH_2_C*H*_3_), 1.30 (3H, t, ^3^*J*_HH_ = 7.0 Hz, OCH_2_C*H*_3_), 1.40 (3H, t, ^3^*J*_HH_ = 6.7 Hz, OCH_2_C*H*_3_), 3.72 (2H, q, ^3^*J*_HH_ = 7.0 Hz, OC*H*_2_CH_3_), 4.23 (2H, q, ^3^*J*_HH_ = 5.5 Hz, OC*H*_2_CH_3_), 4.42 (2H, q, ^3^*J*_HH_ = 5.5 Hz, OC*H*_2_CH_3_), 5.36 (1H, d, ^3^*J*_PH_ = 16.1 Hz, C*H*), 6.80–7.15 (2H, m, Ar*H*), 7.35–7.66 (15H, m, 3 C_6_H_5_), 7.82–7.89 (2H, m, Ar*H*); ^13^C NMR (63.0 MHz, CDCl_3_): δ 8.4 (OCH_2_*C*H_3_), 10.8 (2 OCH_2_*C*H_3_), 30.6 (d, ^1^*J*_PC_ = 135.5 Hz, P = *C*), 44.8 (d, ^2^*J*_PC_ = 20.8 Hz, P = C–*C*H), 58.1 (2 O*C*H_2_CH_3_), 60.1 (O*C*H_2_CH_3_), 108.0 (*C*H_Ar_), 109.6 (*C*H_Ar_), 111.5 (*C*N), 122.1 (*C*H_Ar_), 131.4 (*C*H_Ar_), 121.5 (d, ^1^*J*_PC_ = 125.4 Hz, C_ipso_), 125.4 (d, ^3^*J*_PC_ = 11.03 Hz, C_meta_), 128.9 (C_ortho_), 131.4 (C_para_), 130.4 (*C*_Ar_), 132.6 (*C* = CCO_2_), 128.2 (C = *C*CO_2_), 132.6 (*C*_Ar_), 156.5 (N*C*O), 158.5 (*C*O_2_Et), 161.5 (d, ^3^*J*_PC_ = 11.0 Hz, *C*OCH_2_CH_3_), 163.0 (d, ^2^*J*_PC_ = 12.7 Hz, P = C–*C*O); ^31^P NMR (101.2 MHz, CDCl_3_): δ 21.18 (Ph_3_P^+^–C). Minor isomer: ^1^H NMR (250 MHz, CDCl_3_) δ/ppm: 0.84 (3H, br s, OCH_2_C*H*_3_), 1.30 (3H, t, ^3^*J*_HH_ = 7.0 Hz, OCH_2_C*H*_3_), 1.42 (3H, br s, OCH_2_C*H*_3_), 3.72 (2H, q, ^3^*J*_HH_ = 7.0 Hz, OC*H*_2_CH_3_), 4.23 (2H, q, ^3^*J*_HH_ = 5.5 Hz, OC*H*_2_CH_3_), 4.42 (2H, q, ^3^*J*_HH_ = 5.5 Hz, OC*H*_2_CH_3_), 5.22 (1H, d, ^3^*J*_PH_ = 16.5 Hz, C*H*), 6.80–7.15 (2H, m, Ar*H*), 7.35–7.66 (15H, m, 3 C_6_H_5_), 7.82–7.89 (2H, m, Ar*H*); ^13^C NMR (63.0 MHz, CDCl_3_): δ 8.4 (OCH_2_*C*H_3_), 10.8 (2 OCH_2_*C*H_3_), 30.6 (d, ^1^*J*_PC_ = 135.5 Hz, P = *C*), 44.8 (d, ^2^*J*_PC_ = 20.8 Hz, P = C–*C*H), 58.1 (2 O*C*H_2_CH_3_), 60.1 (O*C*H_2_CH_3_), 108.0 (*C*H_Ar_), 109.6 (*C*H_Ar_), 111.5 (*C*N), 122.1 (*C*H_Ar_), 131.4 (*C*H_Ar_), 121.5 (d, ^1^*J*_PC_ = 125.4 Hz, C_ipso_), 125.4 (d, ^3^*J*_PC_ = 11.03 Hz, C_meta_), 128.9 (C_ortho_), 131.4 (C_para_), 130.4 (*C*_Ar_), 132.6 (*C* = CCO_2_), 128.2 (C = *C*CO_2_), 132.6 (*C*_Ar_), 156.5 (N*C*O), 158.5 (*C*O_2_Et), 161.5 (d, ^3^*J*_PC_ = 11.0 Hz, *C*OCH_2_CH_3_), 163.0 (d, ^2^*J*_PC_ = 12.7 Hz, P = C–*C*O); ^31^P NMR (101.2 MHz, CDCl_3_): δ 22.50 (Ph_3_P^+^–C).

#### *Methyl 5,5-dicyano-2-hydroxy-2',4-dioxo-3-(triphenyl-λ*^*5*^*-phosphanylidene)spiro[cyclopentane-1,3'-indoline]-2-carboxylate (13)*

Dark red powder; Yield (0.5 g, 86%), mp: 96–99 °C; IR (KBr, υ_max_): 3442 (NH), 2229 (C≡N), 1720 (C=O) cm^-1^; MS (*m/z*, %): 585.5 (M^+^, 1), 557.5 (1), 277.3 (100), 262.3 (8), 199.1 (47), 183.2 (38), 77.2 (65). Anal. Calcd for C_34_H_24_N_3_O_5_P (585.6): C, 69.74; H, 4.13; N, 7.18%. Found: C, 69.78; H, 4.09; N, 7.24%. ^1^H NMR (250 MHz, CDCl_3_): δ 3.80 (3H, s, OC*H*_3_), 6.87 (1H, d, ^3^*J* = 6.0 Hz, Ar*H*), 6.92 (1H, s, -O*H*), 7.20 (1H, t, ^3^*J* = 7.5 Hz, Ar*H*), 7.37–7.70 (16H, m, 3 C_6_H_5_ and Ar*H*), 8.01 (1H, d, ^3^*J* = 7.5 Hz, Ar*H*), 10.81 (1H, brs, N*H*); ^13^C NMR (63.0 MHz, CDCl_3_): δ 49.1 (HO*C*CO_2_CH_3_), 51.5 (*C*_spiro_) 53.7 (CO_2_*C*H_3_), 62.2 (*C*(CN)2), 68.3 (d, ^1^*J*_PC_ = 119.5 Hz, P = *C*), 107.5 (*C*N), 109.5 (*C*N), 129.0 (d, ^1^*J*_PC_ = 104.6 Hz, C_ipso_), 125.4 (d, ^3^*J*_PC_ = 12.0 Hz, C_ortho_), 130.0 (C_meta_), 128.8 (C_para_), 108.7 (*C*H_Ar_), 115.5 (*C*_Ar_), 119.8 (*C*H_Ar_), 123.4 (*C*H_Ar_), 134.5 (*C*H_Ar_), 143.6 (*C*_Ar_), 161.5 (HN*C*O), 164.8 (*C*OCH_3_), 180.1(d, ^2^*J*_PC_ = 9.5 Hz, P = C–*C*O); ^31^P NMR (101.2 MHz, CDCl_3_): δ 9.30 (Ph_3_P^+^–C).

#### *Dimethyl 2-(3-(2,4-dioxopentan-3-ylidene)-2-oxoindolin-1-yl)-3-(triphenyl-λ*^*5*^*-phosphanylidene)succinate (15)*

Brown powder; Yield (0.48 g, 76%), mp: 98–101 °C; IR (KBr, υ_max_): 1737 (C = O) cm^-1^; MS (*m/z*, %): 633.5 (M^+^, 1), 517.5 (4), 376.5 (25), 277.3 (35), 262.3 (2), 77.2 (100). Anal. Calcd for C_37_H_32_NO_7_P (633.6): C, 70.14; H, 5.09; N, 2.21%. Found: C, 70.21; H, 5.13; N, 2.32%. Only product: ^1^H NMR (250 MHz, CDCl_3_): δ 2.04 and 2.06 (6H, 2s, OCC*H*_3_), 2.62 and 3.72 (6H, 2s, OC*H*_3_), 5.57 (1H, d, ^3^*J*_PH_ = 16.5 Hz, C*H*), 6.80–8.50 (19H, m, Ar*H* and 3 C_6_H_5_); ^31^P NMR (101.2 MHz, CDCl_3_): δ 20.80 (Ph_3_P^+^–C). Minor isomer: ^1^H NMR (250 MHz, CDCl_3_) δ/ppm: 1.80 and 2.04 (6H, 2s, OCC*H*_3_), 3.08 and 3.40 (6H, 2s, OC*H*_3_), 5.13 (1H, d, ^3^*J*_PH_ = 17.8 Hz, C*H*), 6.80–8.50 (19H, m, Ar*H* and 3 C_6_H_5_); ^31^P NMR (101.2 MHz, CDCl_3_): δ 23.51 (Ph_3_P^+^–C).

#### *Dimethyl 2-(4'-(ethoxycarbonyl)-2,5'-dioxospiro[indoline-3,3'-pyrazolidin]-1-yl)-3-(triphenyl-λ*^*5*^*-phosphanylidene)succinate (16)*

Orange powder; Yield (0.59 g, 88%), mp: 100–103 °C; IR (KBr, υ_max_): 3435 (NH), 1735 (C=O) cm^-1^; MS (*m/z*, %): 649.6 (M^+^-N_2_H_2_, 1), 622.5 (1), 262.3 (100), 183.2 (83), 77.2 (60). Anal. Calcd for C_37_H_34_N_3_O_8_P (679.7): C, 65.39; H, 5.04; N, 6.18%. Found: C, 65.44; H, 4.89; N, 6.26%. Major isomer: ^1^H NMR (250 MHz, CDCl_3_): δ 1.22 (3H, t, ^3^*J*_HH_ = 6.8 Hz, OCH_2_C*H*_3_), 2.01 (1H, s, CH), 3.11 (3H, s, OC*H*_3_), 3.78 (3H, s, OC*H*_3_), 3.80 (2H, q, ^3^*J*_HH_ = 6.8 Hz, OC*H*_2_CH_3_), 5.22 (1H, d, ^3^*J*_PH_ = 15.6 Hz, C*H*), 6.84–7.02 (4H, m, Ar*H*), 7.10–7.68 (15H, m, 3 C_6_H_5_), 9.26 (1H, s, N*H*), 10.44 (1H, s, N*H*); ^13^C NMR (63.0 MHz, CDCl_3_): δ 11.0 (OCH_2_*C*H_3_), 21.7 (*C*H), 28.7 (d, ^1^*J*_PC_ = 124.0 Hz, P = *C*), 49.1 (d, ^2^*J*_PC_ = 18.8 Hz, P = C–*C*H), 50.3 and 51.0 (2 O*C*H_3_), 61.5 (O*C*H_2_CH_3_), 107.5 (*C*H_Ar_), 115.2 (*C*H_Ar_), 118.8 (*C*H_Ar_), 124.6 (*C*H_Ar_), 119.0 (d, ^1^*J*_PC_ = 123.2 Hz, C_ipso_), 125.3 (d, ^3^*J*_PC_ = 12.6 Hz, C_meta_), 127.4 (d, ^2^*J*_PC_ = 9.4 Hz, C_ortho_), 128.2 (*C*_Ar_), 128.9 (C_para_), 132.1 (*C* = CCO_2_), 126.5 (C = *C*CO_2_), 135.6 (*C*_Ar_), 158.1 (N*C*O), 160.0 (*C*O_2_Et), 163.5 (d, ^2^*J*_PC_ = 12.7 Hz, P = C–*C*O), 166.3 (d, ^3^*J*_PC_ = 12.1 Hz, *C*OCH_3_). ^31^P NMR (101.2 MHz, CDCl_3_) δ/ppm: 21.42 (Ph_3_P^+^–C); Minor isomer: ^1^H NMR (250 MHz, CDCl_3_): δ 2.10 (3H, t, ^3^*J*_HH_ = 8.8 Hz, OCH_2_C*H*_3_), 2.87 (1H, s, CH), 3.63 (3H, s, OC*H*_3_), 3.74 (3H, s, OC*H*_3_), 4.15 (2H, m, OC*H*_2_CH_3_), 5.80 (1H, br s, C*H*), 6.84–7.02 (4H, m, Ar*H*), 7.10–7.68 (15H, m, 3 C_6_H_5_), 9.26 (1H, s, N*H*), 10.44 (1H, s, N*H*); ^13^C NMR (63.0 MHz, CDCl_3_): δ 13.7 (OCH_2_*C*H_3_), 22.3 (*C*H), 30.2 (d, ^1^*J*_PC_ = 125.2 Hz, P = *C*), 46.5 (d, ^2^*J*_PC_ = 21.0 Hz, P = C–*C*H), 49.2 and 51.0 (2 O*C*H_3_), 63.0 (O*C*H_2_CH_3_), 106.3 (*C*H_Ar_), 115.6 (*C*H_Ar_), 118.8 (*C*H_Ar_), 126.0 (*C*H_Ar_), 121.5 (d, ^1^*J*_PC_ = 110.7 Hz, C_ipso_), 126.5 (d, ^3^*J*_PC_ = 11.3 Hz, C_meta_), 127.4 (d, ^2^*J*_PC_ = 9.4 Hz, C_ortho_), 128.0 (*C*_Ar_), 128.9 (C_para_), 132.7 (*C* = CCO_2_), 126.5 (C = *C*CO_2_), 133.5 (*C*_Ar_), 158.1 (N*C*O), 160.0 (*C*O_2_Et), 161.7 (d, ^2^*J*_PC_ = 12.0 Hz, P = C–*C*O), 167.5 (d, ^3^*J*_PC_ = 13.5 Hz, *C*OCH_3_); ^31^P NMR (101.2 MHz, CDCl_3_): δ 23.68 (Ph_3_P^+^–C).

### General procedure for the synthesis of phosphonate esters (exemplified by 17)

To a stirred solution of isatin (0.147 g, 1 mmol) and triphenylphosphite (0.31 g, 1 mmol) in 10 mL of CH_2_Cl_2_, a mixture of dimethyl acetylenedicarboxylate (0.142 g, 1 mmol) in 3 mL of CH_2_Cl_2_ was added drop-wise at room temperature over 10 min. The mixture was then allowed to stir for 24 h. The solvent was removed through slow evaporation, and the remaining substance was washed with diethyl ether to obtain the crude adducts.

#### Dimethyl 2-(2,3-dioxoindolin-1-yl)-3-(diphenoxyphosphanyl)succinate (*17*)

Orange powder; Yield (0.47 g, 90%), mp: 112–114 °C; IR (KBr, υ_max_): 1731 (C=O), 1615 cm^-1^ ^42^.

#### Dimethyl 2-[3-(1-cyano-2-ethoxy-2-oxoethylidene)-2-oxoindolin-1-yl]-3-(diphenoxyphosphanyl)succinate (*18*)

Dark red powder; Yield (0.48 g, 78%), mp: 89–91 °C; IR (KBr, υ_max_): 2216 (C≡N), 1745, 1726, 1615 (C=O) cm^-1^; MS (*m/z*, %): 619.5 (M^+^ + 1, 5), 618.5 (M^+^, 2), 573.4 (2), 525.3 (35), 430.2 (58), 241 (10), 223.0 (61), 76.9 (100). Anal. Calcd for C_31_H_27_N_2_O_10_P (618.5): C, 60.20; H, 4.40; N, 4.53%. Found: C, 60.28; H, 4.34; N, 4.61%. ^1^H NMR (250 MHz, CDCl_3_): δ 1.44 (3H, t, *J* = 7.0 Hz, OCH_2_C*H*_3_), 3.75 (3H, s, OC*H*_3_), 3.83–4.30 (1H, m, PC*H*CH), 3.90 (3H, s, OC*H*_3_), 4.46 (2H, q, *J* = 7.0 Hz, OC*H*_2_CH_3_), 5.61 (1H, brs, PCHC*H*), 6.90–7.44 (10H, m, 2 OC_6_*H*_5_), 7.63 (1H, t, *J* = 8.0 Hz, Ar*H*), 8.30 (1H, d, *J* = 7.5 Hz, Ar*H*), 8.52 (1H, brs, Ar*H*), 8.63 (1H, brs, Ar*H*); ^13^C NMR (63.0 MHz, CDCl_3_): δ 10.8 (OCH_2_*C*H_3_), 46.0 (d, ^1^*J*_PC_ = 118.0 Hz, P*C*HCH), 45.6 (PCH*C*H), 50.27 and 52.03 (2s, 2 O*C*H_3_), 60.24 (O*C*H_2_CH_3_), 107.9 (C*H*_Ar_), 116.03 (CN), 109.5 (C*H*_Ar_), 110.9 (C = *C*CO_2_), 116.9 (*C*_Ar_), 120.0 and 120.8 (2s, 4C_ortho_), 126.6 and 126.9 (2s, 2C_para_), 146.3 (d, ^2^*J*_PC_ = 15.1 Hz, 2C_ipso_), 132.8 and 135.5 (4C_meta_), 128.4 (*C* = CCO_2_), 132.8 (*C*H_Ar_), 135.5 (*C*H_Ar_), 146.2 (*C*_Ar_), 156.4 (N*C*O), 161.5 (*C*O_2_Et), 156.4 (*C*OCH_3_), 162.7 (P = C–*C*O); ^31^P NMR (101.2 MHz, CDCl_3_): δ 10.04 (O = P(OPh)_2_).

## Conclusion

In summary, we have demonstrated that 2-oxoindolin-3-ylidene derivatives can serve as an important heterocyclic core for synthesizing previously unreported phosphorus ylides and phosphonate esters. The newly synthesized organophosphorus compounds were produced under mild reaction conditions and may possess high chemical and biological properties. Merging the phosphorus ylide moiety with high-potential biologically active structures could be more interesting for scientists.

### Supplementary Information

Supplementary Information.

## Data Availability

All data generated or analysed during this study are included in this published article and its supplementary information files.
