# Supplementary material for: Synthesis of novel organophosphorus compounds via reaction of substituted 2-oxoindoline-3-ylidene with acetylenic diesters and triphenylphosphine or triphenyl phosphite
Source: Sci Rep. 2024 Mar 15;14:6314. doi: 10.1038/s41598-024-56774-z (PMC10943016; doi:10.1038/s41598-024-56774-z)
Supplement: Supplementary file 1 — Supplementary Information. [file 41598_2024_56774_MOESM1_ESM.pdf]

Supplementary data for

Synthesis of novel organophosphorus compounds *via* reaction of substituted 2-oxoindoline-3-ylidene with acetylenic diesters and triphenylphosphine or triphenyl phosphite

Mahsa Najafi, Ghasem Marandi

Department of Organic Chemistry, Faculty of Chemistry, Urmia University, Urmia, Iran

Contents

1- **General information** ……………………………………………………………..2
2- Experimental procedures……………….………………………………………..2
3- ^{1}H and ^{13}C NMR spectra of the products……..…………………………………..7

Experimental

All melting points were measured using a Barnstead Electrothermal 9200 apparatus. In addition, IR spectra of the synthesized compounds were recorded with a Thermo-Nicolet Nexus 670 FT-IR spectrometer. The ^{1}H, ^{13}C and ^{31}P NMR spectra for the obtained compounds were recorded using a BRUKER DRX-250 AVANCE instruments with $CDCl_3$ as the solvent and TMS as the internal standard at frequencies of (250.1, 62.9 and 101.3) MHz, respectively. The mass spectra of newly synthesized compounds were analyzed using an Agilent 5975C mass spectrometer operating at an ionization potential of 70 eV. Elemental analyses (C, H, N) were conducted using a Heraeus CHN-O-Rapid analyzer. Triphenylphosphine, triphenylphosphite, acetylenic esters, ethylcyanoacetate, sodium azide, isatin, thiazolidine-2,4-dione, malononitrile and, acetyl acetone as well as all solvents were purchased from Merck, Fluka and Sigma-Aldrich companies and used without additional purification.

General procedure for the synthesis of NH source compounds (exemplified by 4) To a magnetically stirred solution of ethyl cyanoacetate (0.113 g, 1 mmol) and isatin (0.147 g, 1 mmol) in EtOH (10 mL) was prepared, then a mixture of sodium azide (0.07 g, 1.1 mmol) in EtOH (5 mL) was added dropwise over 5 minutes at room temperature. Then, the mixture was heated to 70 °C for 10 hours to complete the reaction, which was monitored by TLC). The solvent was removed through slow evaporation. All residues were washed with cold diethyl ether (2×3 mL), and the desired product was then filtered and recrystallized from ethanol (3 mL).

*Ethyl 2-(2-oxoindolin-3-ylidene)-2-(2H-tetrazol-5-yl)acetate (**4**)*

Brown powder, Yield (0.23 g, 81%), mp: 126-128 °C; IR (KBr, υ_{max}): 3439 (NH_{tet}), 3370 (NH_{isat}), 1718 (C=O) cm^{-1}; ^{1}H NMR (250 MHz, $CDCl_3$): δ 1.44 (3H, t, *J*= 7 Hz, OCH_2CH_3), 4.46 (2H, q, *J*= 7 Hz, OCH_2CH_3), 6.89 (1H, d, *J*= 8 Hz, Ar*H*), 7.04 (1H, t, *J*= 7.5 Hz, Ar*H*), 7.43 (1H, t, *J*= 8.0 Hz, Ar*H*), 7.80 (1H, brs, N*H*), 8.32 (1H, d, *J*= 8.0 Hz, Ar*H*). ^{13}C NMR (63.0 MHz, $CDCl_3$): δ 13.9 (OCH_2CH_3), 63.4 (OCH_2CH_3), 111.0 (CH_{Ar}), 123.2 (C_{Ar}), 124.0 (*C*=CCO), 125.7 (CH_{Ar}), 130.1 (CH_{Ar}), 135.9 (CH_{Ar}), 138.7 (C=*C*CO), 144.1 (HNC_{Ar}), 145.0 (C_{tet}), 150.0 (HN*C*O), 166.5 (CO_2Et).

General Procedure for the Synthesis of phosphorus ylides (Exemplified by 6)

To a magnetically stirred solution of ethyl 2-(2-oxoindolin-3-ylidene)-2-(2*H*-tetrazol-5-yl)acetate **4** (0.285 g, 1 mmol) and triphenylphosphine (0.262 g, 1 mmol) in ethyl acetate (10 mL), dimethyl acetylenedicarboxylate (0.142 g, 1m mol) in ethyl acetate (3 mL) was added dropwise at room temperature. After approximately 24 hours of stirring at room temperature, the crude products were collected and washed with cold diethyl ether (2×3 mL).

*Dimethyl 2-(3-(2-ethoxy-2-oxo-1-(2H-tetrazol-5-yl)ethylidene)-2-oxoindolin-1-yl)-3-(triphenyl-λ^5-phosphanylidene)succinate (**6**)*

Red powder, Yield (0.54 g, 78%), mp: 78-81 °C; IR (KBr, υ_{max}): 3448 (NH_{tet}), 1735 (C=O) cm^{-1}; MS (*m/z*, %): 689.6 (M^+, 1), 557.5 (4), 427.4 (1), 277.2 (100), 262.3 (49), 183.1 (58), 77.1 (62). Anal. Calcd for $C_{37}H_{32}N_5O_7P$ (689.7): C, 64.44; H, 4.68; N, 10.15%. Found: C, 64.61; H, 4.52; N, 10.27%. Major isomer: ^{1}H NMR (250 MHz, $CDCl_3$): δ 1.40 (3H, brs, OCH_2CH_3), 3.11 (3H, s, OCH_3), 3.77 (3H, s, OCH_3), 4.33 (2H, brs, OCH_2CH_3), 5.36 (1H, d, $^{3}J_{PH}$= 18.3 Hz, C*H*), 6.84-7.10 (3H, m, Ar*H*), 7.45-7.80 (15H, m, 3 C_6H_5),

8.58-8.18 (1H, brs, ArH); ^{13}C NMR (63.0 MHz, $CDCl_3$): δ 10.8 (OCH_2CH_3), 37.3 (d, $^1J_{PC}$= 120.3 Hz, P=*C*), 49.1 and 49.6 (2s, 2 O*C*H_3), 51.5 (P=C-*C*H, d, $^2J_{PC}$= 15.1 Hz), 60.0 (O*C*H_2CH_3), 125.4 (d, $^3J_{PC}$=12.0 Hz, C_{meta}), 126.0 (d, $^1J_{PC}$=82.0 Hz, C_{ipso}), 128.9 (d, $^2J_{PC}$=7.0 Hz, C_{ortho}), 132.6 (C_{para}), 108.0 (*C*H_{Ar}), 109.6 (*C*$_{Ar}$), 125.7 (*C*H_{Ar}), 130.2 (*C*H_{Ar}), 131.4 (*C*=CCO_2), 132.6 (C=*C*CO_2), 135.2 (*C*H_{Ar}), 147.4 (*C*$_{Ar}$), 158.8 (N*C*O), 156.3 (*C*$_{tet}$), 162.3 (*C*O_2Et), 166.8 (d, $^3J_{PC}$= 11.5 Hz, *C*OCH_3), 167.7 (d, $^2J_{PC}$= 14.7 Hz, P=C-*C*O); ^{31}P NMR (101.2 MHz, $CDCl_3$): δ 22.43 (Ph_3P^+–C); Minor isomer: ^{1}H NMR (250 MHz, $CDCl_3$): δ 1.28 (3H, brs, OCH_2CH_3), 3.77 (3H, s, OC*H*3), 4.23 (2H, brs, OCH_2CH_3), 4.42 (3H, s, OCH_3), 5.23 (1H, d, $^3J_{PH}$= 17.8 Hz, C*H*), 6.84-7.10 (3H, m, Ar*H*), 7.45-7.80 (15H, m, 3 C_6H_5), 8.58-8.18 (1H, brs, ArH). ^{13}C NMR (63.0 MHz, $CDCl_3$) δ/ppm: 10.5 (OCH_2CH_3), 36.0 (d, $^1J_{PC}$= 122.1 Hz, P=*C*), 48.9 and 49.4 (2s, 2 O*C*H_3), 47.5 (P=C-*C*H, d, $^2J_{PC}$= 22.1 Hz), 60.0 (O*C*H_2CH_3), 125.8 (d, $^3J_{PC}$=12.0 Hz, C_{meta}), 127.4 (d, $^1J_{PC}$=104.0 Hz, C_{ipso}), 128.9 (d, $^2J_{PC}$=7.0 Hz, C_{ortho}), 130.2 (C_{para}), 110.8 (*C*H_{Ar}), 111.0 (*C*$_{Ar}$), 121.7 (*C*HAr), 130.8 (*C*=CCO), 128.1 (*C*H_{Ar}), 132.6 (*C*H_{Ar}), 131.0 (C=*C*CO), 146.7 (*C*$_{Ar}$), 159.0 (N*C*O), 158.5 (*C*$_{tet}$), 164.5 (*C*O_2Et), 166.4 (d, $^3J_{PC}$= 12.3 Hz, *C*OCH_3), 167.5 (d, $^2J_{PC}$= 13.8 Hz, P=C-*C*O); ^{31}P NMR (101.2 MHz, $CDCl_3$) δ/ppm: 22.78 (Ph_3P^+–C).

*Dimethyl 2-[2,4-dioxo-5-(2-oxoindolin-3-ylidene)thiazolidin-3-yl]-3-(triphenyl-λ⁵-phosphanylidene)succinate (**10**)*

Orange powder, Yield (0.49 g, 75%), mp: 80-83 °C; IR (KBr, υ_{max}): 3420 (NH), 1738 (C=O) cm^{-1}; MS (*m/z*, %): 650.6 (M^+, 1), 557.6 (11), 388.3 (4), 277.3 (100), 246.3 (17), 262.3 (37), 183.1 (28), 77.1 (32). Anal. Calcd for $C_{35}H_{27}N_2O_7PS$ (650.6): C, 64.61; H, 4.18; N, 4.31%. Found: C, 64.77; H, 4.13; N, 4.36%. Major isomer: ^{1}H NMR (250 MHz, $CDCl_3$): δ 3.11 (3H, s, OCH_3), 3.79 (3H, s, OCH_3), 5.31 (1H, d, $^3J_{PH}$= 16.0 Hz, C*H*), 6.85-7.82 (4H, m, Ar*H*), 7.45-7.82 (15H, m, 3 C_6H_5), 9.76 (1H, brs, N*H*); ^{13}C NMR (63.0 MHz, $CDCl_3$): δ 33.2 (d, $^1J_{PC}$= 100.0 Hz, P=*C*), 46.4 and 49.1 (2s, 2 O*C*H_3), 51.5 (P=C-*C*H, d, $^2J_{PC}$= 15.1 Hz), 125.4 (d, $^3J_{PC}$=11.3 Hz, C_{meta}), 127.3 (d, $^1J_{PC}$=91.4 Hz, C_{ipso}), 128.9 (C_{ortho}), 130.2 (C_{para}), 109.6 (*C*H_{Ar}), 111.2 (*C*$_{Ar}$), 120.1 (*C*H_{Ar}), 122.2 (*C*H_{Ar}), 129.3 (C=*C*SCO), 130.2 (*C*=CSCO), 135.3 (*C*H_{Ar}), 147.2 (*C*$_{Ar}$), 156.3 (HN*C*O), 166.3 (d, $^3J_{PC}$= 11.2 Hz, *C*OCH_3), 167.5 (d, $^2J_{PC}$= 13.8 Hz, P=C-*C*O), 173.0 (*C*ONCOS), 180.9 (CON*C*OS); ^{31}P NMR (101.2 MHz, $CDCl_3$): δ 19.45 (Ph_3P^+–C); Minor isomer: ^{1}H NMR (250 MHz, $CDCl_3$) δ/ppm: 3.70 (3H, s, OCH_3), 3.79 (3H, s, OCH_3), 5.23 (1H, d, $^3J_{PH}$= 16.0 Hz, C*H*), 6.85-7.82 (4H, m, Ar*H*), 7.45-7.82 (15H, m, 3 C_6H_5), 9.76 (1H, brs, N*H*); ^{13}C NMR (63.0 MHz, $CDCl_3$): δ 36.3 (d, $^1J_{PC}$= 107.9 Hz, P=*C*), 49.1 and 49.7 (2s, 2 O*C*H_3), 52.6 (P=C-*C*H, d, $^2J_{PC}$= 15.0 Hz), 125.9 (d, $^3J_{PC}$=11.3 Hz, C_{meta}), 127.3 (d, $^1J_{PC}$=91.4 Hz, C_{ipso}), 129.3 (C_{ortho}), 130.2 (C_{para}), 107.8 (C_{Ar}), 114.7 (*C*H_{Ar}), 121.4 (*C*H_{Ar}), 123.0 (*C*H_{Ar}), 129.5 (C=*C*SCO), 130.2 (*C*=CSCO), 138.5 (*C*H_{Ar}), 147.2 (*C*$_{Ar}$), 153.6 (HN*C*O), 166.7 (d, $^2J_{PC}$= 13.0 Hz, P=C-*C*O), 169.8 (d, $^3J_{PC}$= 11.5 Hz, COCH3), 173.0 (*C*ONCOS), 181.2 (CON*C*OS); ^{31}P NMR (101.2 MHz, $CDCl_3$): δ 19.63 (Ph_3P^+–C).

*Dimethyl 2-[3-(1-cyano-2-ethoxy-2-oxoethylidene)-2-oxoindolin-1-yl]-4-(methylperoxy)-3-(triphenyl-λ⁵-phosphanylidene)butanoate (**11**)*

Dark red powder; Yield (0.56 g, 86%), mp: 82-85 °C; IR (KBr, υ_{max}): 2200 (C≡N), 1750 and 1720 (C=O) cm^{-1}; MS (*m/z*, %): 646.5 (M^+, 1), 384.5 (1), 355.4 (2), 277.3 (100), 262.3 (8), 185.2 (47), 77.2 (65). Anal. Calcd for $C_{37}H_{31}N_2O_7P$ (646.6): C, 68.73; H, 4.83; N, 4.33%. Found: C, 68.80; H, 4.72; N, 4.41%. Major isomer: ^{1}H NMR (250 MHz, $CDCl_3$): δ 1.43 (3H, t, $^3J_{HH}$= 6.8 Hz, OCH_2CH_3), 3.12 (3H, s, OCH_3), 3.77 (3H, s, OCH_3), 4.38 (2H, q, $^3J_{HH}$= 6.8 Hz, OCH_2CH_3), 5.37 (1H, d, $^3J_{PH}$= 16.3 Hz, C*H*), 7.10 (1H, t, ^3J= 7.0 Hz, Ar*H*), 7.35-7.73 (15H, m, 3 C_6H_5), 7.80 (1H, brs, Ar*H*), 8.00 (1H, brs, Ar*H*), 8.18 (1H, d, ^3J= 7.5 Hz, Ar*H*); ^{13}C NMR (63.0 MHz, $CDCl_3$): δ 14.0 (OCH_2CH_3), 29.5 (d, $^1J_{PC}$= 122.1 Hz, P=*C*), 52.3 and 54.4 (2s, 2 O*C*H_3), 54.5 (P=C-*C*H, d, $^2J_{PC}$= 13.5 Hz), 63.3 (O*C*H_2CH_3), 124.5 (d, $^1J_{PC}$=120.3 Hz, C_{ipso}), 128.5 (d, $^3J_{PC}$=12.0 Hz, C_{meta}), 132.0 (C_{ortho}, C_{para}), 111.0 (*C*H_{Ar}), 114.1 (*C*$_{Ar}$), 125.5 (*C*H_{Ar}), 130.0 (*C*H_{Ar}), 133.4 (*C*=CCO_2), 135.8 (C=*C*CO_2), 134.6 (*C*H_{Ar}), 145.5 (*C*$_{Ar}$), 152.5 (N*C*O), 166.3 (*C*O_2Et), 163.5 (d, $^3J_{PC}$= 12.0

Hz, *C*OCH$_3$), 168.0 (d, $^2J_{PC}$= 13.7 Hz, P=C-*C*O); ^{31}P NMR (101.2 MHz, CDCl$_3$): δ 22.42 (Ph$_3$P$^+$–C); Minor isomer: ^{1}H NMR (250 MHz, CDCl$_3$): δ 1.23 (3H, uneven t, OCH$_2$C*H*$_3$), 3.49 (3H, s, OC*H*$_3$), 3.68 (3H, s, OC*H*$_3$), 4.30 (2H, m, OC*H*$_2$CH$_3$), 5.37 (1H, d, $^3J_{PH}$= 16.3 Hz, C*H*), 7.10 (1H, t, ^3J= 7.0 Hz, Ar*H*), 7.35-7.73 (15H, m, 3 C$_6$H$_5$), 7.80 (1H, brs, Ar*H*), 8.00 (1H, brs, Ar*H*), 8.18 (1H, d, ^3J= 7.5 Hz, Ar*H*); ^{13}C NMR (63.0 MHz, CDCl$_3$): δ 13.3 (OCH$_2$*C*H$_3$), 32.7 (d, $^1J_{PC}$= 119.5 Hz, P=*C*), 51.0 and 53.2 (2s, 2 O*C*H$_3$), 54.5 (P=C-*C*H, d, $^2J_{PC}$= 13.5 Hz), 64.6 (O*C*H$_2$CH$_3$), 124.5 (d, $^1J_{PC}$=120.3 Hz, C$_{ipso}$), 128.9 (d, $^3J_{PC}$=11.3 Hz, C$_{meta}$), 132.3 (C$_{ortho}$, C$_{para}$), 112.6 (*C*H$_{Ar}$), 114.3 (*C*$_{Ar}$), 125.5 (*C*H$_{Ar}$), 130.0 (*C*H$_{Ar}$), 133.4 (*C*=CCO$_2$), 135.8 (C=*C*CO$_2$), 134.6 (*C*H$_{Ar}$), 145.5 (*C*$_{Ar}$), 153.1 (N*C*O), 166.3 (*C*O$_2$Et), 164.6 (d, $^3J_{PC}$= 12.0 Hz, *C*OCH$_3$), 170.1 (d, $^2J_{PC}$= 13.7 Hz, P=C-*C*O); ^{31}P NMR (101.2 MHz, CDCl$_3$): δ 22.75 (Ph$_3$P$^+$–C).

*Diethyl 2-(3-(1-cyano-2-ethoxy-2-oxoethylidene)-2-oxoindolin-1-yl)-3-(triphenyl-λ^5-phosphanylidene)succinate (**12**)*

Dark red powder; Yield (54 g, 80%), mp: 94-97 °C; IR (KBr, υ$_{max}$): 2230 (C≡N), 1735 (C=O) cm^{-1}; MS (*m/z*, %): 674.8 (M$^+$, 1), 647.5 (1), 563.1 (1), 412.4 (10), 277.3 (86), 262.3 (100), 183.2 (98), 77.1 (43). Anal. Calcd for C$_{39}$H$_{37}$N$_2$O$_7$P (676.7): C, 69.22; H, 5.51; N, 4.14%. Found: C, 69.31; H, 5.47; N, 4.22%. Major isomer: ^{1}H NMR (250 MHz, CDCl$_3$): δ 0.44 (3H, br s, OCH$_2$C*H*$_3$), 1.30 (3H, t, $^3J_{HH}$= 7.0 Hz, OCH$_2$C*H*$_3$), 1.40 (3H, t, $^3J_{HH}$= 6.7 Hz, OCH$_2$C*H*$_3$), 3.72 (2H, q, $^3J_{HH}$= 7.0 Hz, OC*H*$_2$CH$_3$), 4.23 (2H, q, $^3J_{HH}$= 5.5 Hz, OC*H*$_2$CH$_3$), 4.42 (2H, q, $^3J_{HH}$= 5.5 Hz, OC*H*$_2$CH$_3$), 5.36 (1H, d, $^3J_{PH}$= 16.1 Hz, C*H*), 6.80-7.15 (2H, m, Ar*H*), 7.35-7.66 (15H, m, 3 C$_6$H$_5$), 7.82-7.89 (2H, m, Ar*H*); ^{13}C NMR (63.0 MHz, CDCl$_3$): δ 8.4 (OCH$_2$*C*H$_3$), 10.8 (2 OCH$_2$*C*H$_3$), 30.6 (d, $^1J_{PC}$= 135.5 Hz, P=*C*), 44.8 (d, $^2J_{PC}$= 20.8 Hz, P=C-*C*H), 58.1 (2 O*C*H$_2$CH$_3$), 60.1 (O*C*H$_2$CH$_3$), 108.0 (*C*H$_{Ar}$), 109.6 (*C*H$_{Ar}$), 111.5 (*C*N), 122.1 (*C*H$_{Ar}$), 131.4 (*C*H$_{Ar}$), 121.5 (d, $^1J_{PC}$=125.4 Hz, C$_{ipso}$), 125.4 (d, $^3J_{PC}$=11.03 Hz, C$_{meta}$), 128.9 (C$_{ortho}$), 131.4 (C$_{para}$), 130.4 (*C*$_{Ar}$), 132.6 (*C*=CCO$_2$), 128.2 (C=*C*CO$_2$), 132.6 (*C*$_{Ar}$), 156.5 (N*C*O), 158.5 (*C*O$_2$Et), 161.5 (d, $^3J_{PC}$= 11.0 Hz, *C*OCH$_2$CH$_3$), 163.0 (d, $^2J_{PC}$= 12.7 Hz, P=C-*C*O); ^{31}P NMR (101.2 MHz, CDCl$_3$): δ 21.18 (Ph$_3$P$^+$–C). Minor isomer: ^{1}H NMR (250 MHz, CDCl$_3$) δ/ppm: 0.84 (3H, br s, OCH$_2$C*H*$_3$), 1.30 (3H, t, $^3J_{HH}$= 7.0 Hz, OCH$_2$C*H*$_3$), 1.80 (3H, br s, OCH$_2$C*H*$_3$), 3.72 (2H, q, $^3J_{HH}$= 7.0 Hz, OC*H*$_2$CH$_3$), 4.23 (2H, q, $^3J_{HH}$= 5.5 Hz, OC*H*$_2$CH$_3$), 4.42 (2H, q, $^3J_{HH}$= 5.5 Hz, OC*H*$_2$CH$_3$), 5.22 (1H, d, $^3J_{PH}$= 16.5 Hz, C*H*), 6.80-7.15 (2H, m, Ar*H*), 7.35-7.66 (15H, m, 3 C$_6$H$_5$), 7.82-7.89 (2H, m, Ar*H*); ^{13}C NMR (63.0 MHz, CDCl$_3$): δ 8.4 (OCH$_2$*C*H$_3$), 10.8 (2 OCH$_2$*C*H$_3$), 30.6 (d, $^1J_{PC}$= 135.5 Hz, P=*C*), 44.8 (d, $^2J_{PC}$= 20.8 Hz, P=C-*C*H), 58.1 (2 O*C*H$_2$CH$_3$), 60.1 (O*C*H$_2$CH$_3$), 108.0 (*C*H$_{Ar}$), 109.6 (*C*H$_{Ar}$), 111.5 (*C*N), 122.1 (*C*H$_{Ar}$), 131.4 (*C*H$_{Ar}$), 121.5 (d, $^1J_{PC}$=125.4 Hz, C$_{ipso}$), 125.4 (d, $^3J_{PC}$=11.03 Hz, C$_{meta}$), 128.9 (C$_{ortho}$), 131.4 (C$_{para}$), 130.4 (*C*$_{Ar}$), 132.6 (*C*=CCO$_2$), 128.2 (C=*C*CO$_2$), 132.6 (*C*$_{Ar}$), 156.5 (N*C*O), 158.5 (*C*O$_2$Et), 161.5 (d, $^3J_{PC}$= 11.0 Hz, *C*OCH$_2$CH$_3$), 163.0 (d, $^2J_{PC}$= 12.7 Hz, P=C-*C*O); ^{31}P NMR (101.2 MHz, CDCl$_3$): δ 22.50 (Ph$_3$P$^+$–C).

*Methyl 5,5-dicyano-2-hydroxy-2',4-dioxo-3-(triphenyl-λ^5-phosphanylidene)spiro[cyclopentane-1,3'-indoline]-2-carboxylate (**13**)*

Dark red powder; Yield (0.5 g, 86%), mp: 96-99 °C; IR (KBr, υ$_{max}$): 3442 (NH), 2229 (C≡N), 1720 (C=O) cm^{-1}; MS (*m/z*, %): 585.5 (M$^+$, 1), 557.5 (1), 277.3 (100), 262.3 (8), 199.1 (47), 183.2 (38), 77.2 (65). Anal. Calcd for C$_{34}$H$_{24}$N$_3$O$_5$P (585.6): C, 69.74; H, 4.13; N, 7.18%. Found: C, 69.78; H, 4.09; N, 7.24%. ^{1}H NMR (250 MHz, CDCl$_3$): δ 3.80 (3H, s, OC*H*$_3$), 6.87 (1H, d, ^3J= 6.0 Hz, Ar*H*), 6.92 (1H, s, -O*H*), 7.20 (1H, t, ^3J= 7.5 Hz, Ar*H*), 7.37-7.70 (16H, m, 3 C$_6$H$_5$ and Ar*H*), 8.01 (1H, d, ^3J= 7.5 Hz, Ar*H*), 10.81 (1H, brs, N*H*); ^{13}C NMR (63.0 MHz, CDCl$_3$): δ 49.1 (HO*C*CO$_2$CH$_3$), 51.5 (*C*$_{spiro}$) 53.7 (CO$_2$*C*H$_3$), 62.2 (*C*(CN)2), 68.3 (d, $^1J_{PC}$= 119.5 Hz, P=*C*), 107.5 (*C*N), 109.5 (*C*N), 129.0 (d, $^1J_{PC}$=104.6 Hz, C$_{ipso}$), 125.4 (d, $^3J_{PC}$=12.0 Hz, C$_{ortho}$), 130.0 (C$_{meta}$), 128.8 (C$_{para}$), 108.7 (*C*H$_{Ar}$), 115.5 (*C*$_{Ar}$), 119.8 (*C*H$_{Ar}$), 123.4 (*C*H$_{Ar}$), 134.5 (*C*H$_{Ar}$), 143.6 (*C*$_{Ar}$), 161.5 (HN*C*O), 164.8 (*C*OCH$_3$), 180.1(d, $^2J_{PC}$= 9.5 Hz, P=C-*C*O); ^{31}P NMR (101.2 MHz, CDCl$_3$): δ 9.30 (Ph$_3$P$^+$–C).

*Dimethyl 2-(3-(2,4-dioxopentan-3-ylidene)-2-oxoindolin-1-yl)-3-(triphenyl-λ⁵-phosphanylidene)succinate (**15**)*

Brown powder; Yield (0.48 g, 76%), mp: 98-101 °C; IR (KBr, υ_{max}): 1737 (C=O) cm^{-1}; MS (*m/z*, %): 633.5 (M^+, 1), 517.5 (4), 376.5 (25), 277.3 (35), 262.3 (2), 77.2 (100). Anal. Calcd for $C_{37}H_{32}NO_7P$ (633.6): C, 70.14; H, 5.09; N, 2.21%. Found: C, 70.21; H, 5.13; N, 2.32%. Only product: ^1H NMR (250 MHz, $CDCl_3$): δ 2.04 and 2.06 (6H, 2s, $OCCH_3$), 2.62 and 3.72 (6H, 2s, OCH_3), 5.57 (1H, d, $^3J_{PH}$= 16.5 Hz, C*H*), 6.80-8.50 (19H, m, Ar*H* and 3 C_6H_5); ^{31}P NMR (101.2 MHz, $CDCl_3$): δ 20.80 (Ph_3P^+–C). Minor isomer: ^1H NMR (250 MHz, $CDCl_3$) δ/ppm: 1.80 and 2.04 (6H, 2s, $OCCH_3$), 3.08 and 3.40 (6H, 2s, OCH_3), 5.13 (1H, d, $^3J_{PH}$= 17.8 Hz, C*H*), 6.80-8.50 (19H, m, Ar*H* and 3 C_6H_5); ^{31}P NMR (101.2 MHz, $CDCl_3$): δ 23.51 (Ph_3P^+–C).

*Dimethyl 2-(4'-(ethoxycarbonyl)-2,5'-dioxospiro[indoline-3,3'-pyrazolidin]-1-yl)-3-(triphenyl-λ⁵-phosphanylidene)succinate (**16**)*

Orange powder; Yield (0.59 g, 88%), mp: 100-103 °C; IR (KBr, υ_{max}): 3435 (NH), 1735 (C=O) cm^{-1}; MS (*m/z*, %): 649.6 (M^+-N_2H_2, 1), 622.5 (1), 262.3 (100), 183.2 (83), 77.2 (60). Anal. Calcd for $C_{37}H_{34}N_3O_8P$ (679.7): C, 65.39; H, 5.04; N, 6.18%. Found: C, 65.44; H, 4.89; N, 6.26%. Major isomer: ^1H NMR (250 MHz, $CDCl_3$): δ 1.22 (3H, t, $^3J_{HH}$= 6.8 Hz, OCH_2CH_3), 2.01 (1H, s, CH), 3.11 (3H, s, OCH_3), 3.78 (3H, s, OCH_3), 3.80 (2H, q, $^3J_{HH}$= 6.8 Hz, OCH_2CH_3), 5.22 (1H, d, $^3J_{PH}$= 15.6 Hz, C*H*), 6.84-7.02 (4H, m, Ar*H*), 7.10-7.68 (15H, m, 3 C_6H_5), 9.26 (1H, s, N*H*), 10.44 (1H, s, N*H*); ^{13}C NMR (63.0 MHz, $CDCl_3$): δ 11.0 (OCH_2CH_3), 21.7 (*C*H), 28.7 (d, $^1J_{PC}$= 124.0 Hz, P=*C*), 49.1 (d, $^2J_{PC}$= 18.8 Hz, P=C-*C*H), 50.3 and 51.0 (2 O*C*H_3), 61.5 (OCH_2CH_3), 107.5 (CH_{Ar}), 115.2 (CH_{Ar}), 118.8 (CH_{Ar}), 124.6 (CH_{Ar}), 119.0 (d, $^1J_{PC}$=123.2 Hz, C_{ipso}), 125.3 (d, $^3J_{PC}$=12.6 Hz, C_{meta}), 127.4 (d, $^2J_{PC}$=9.4 Hz, C_{ortho}), 128.2 (C_{Ar}), 128.9 (C_{para}), 132.1 (*C*=CCO_2), 126.5 (C=*C*CO_2), 135.6 (C_{Ar}), 158.1 (N*C*O), 160.0 (*C*O_2Et), 163.5 (d, $^2J_{PC}$= 12.7 Hz, P=C-*C*O), 166.3 (d, $^3J_{PC}$= 12.1 Hz, *C*OCH_3). ^{31}P NMR (101.2 MHz, $CDCl_3$) δ/ppm: 21.42 (Ph_3P^+–C); Minor isomer: ^1H NMR (250 MHz, $CDCl_3$): δ 2.10 (3H, t, $^3J_{HH}$= 8.8 Hz, OCH_2CH_3), 2.87 (1H, s, CH), 3.63 (3H, s, OCH_3), 3.74 (3H, s, OCH_3), 4.15 (2H, m, OCH_2CH_3), 5.80 (1H, br s, C*H*), 6.84-7.02 (4H, m, Ar*H*), 7.10-7.68 (15H, m, 3 C_6H_5), 9.26 (1H, s, N*H*), 10.44 (1H, s, N*H*); ^{13}C NMR (63.0 MHz, $CDCl_3$): δ 13.7 (OCH_2CH_3), 22.3 (*C*H), 30.2 (d, $^1J_{PC}$= 125.2 Hz, P=*C*), 46.5 (d, $^2J_{PC}$= 21.0 Hz, P=C-*C*H), 49.2 and 51.0 (2 O*C*H_3), 63.0 (OCH_2CH_3), 106.3 (CH_{Ar}), 115.6 (CH_{Ar}), 118.8 (CH_{Ar}), 126.0 (CH_{Ar}), 121.5 (d, $^1J_{PC}$=110.7 Hz, C_{ipso}), 126.5 (d, $^3J_{PC}$=11.3 Hz, C_{meta}), 127.4 (d, $^2J_{PC}$=9.4 Hz, C_{ortho}), 128.0 (C_{Ar}), 128.9 (C_{para}), 132.7 (*C*=CCO_2), 126.5 (C=*C*CO_2), 133.5 (C_{Ar}), 158.1 (N*C*O), 160.0 (*C*O_2Et), 161.7 (d, $^2J_{PC}$= 12.0 Hz, P=C-*C*O), 167.5 (d, $^3J_{PC}$= 13.5 Hz, *C*OCH_3); ^{31}P NMR (101.2 MHz, $CDCl_3$): δ 23.68 (Ph_3P^+–C).

General Procedure for the Synthesis of phosphonate esters (Exemplified by 17)

To a stirred solution of isatin (0.147 g, 1 mmol) and triphenylphosphite (0.31 g, 1 mmol) in 10 mL of CH_2Cl_2, a mixture of dimethyl acetylenedicarboxylate (0.142 g, 1 mmol) in 3 mL of CH_2Cl_2 was added drop-wise at room temperature over 10 min. The mixture was then allowed to stir for 24 hours. The solvent was removed through slow evaporation, and the remaining substance was washed with diethyl ether to obtain the crude adducts.

*Dimethyl 2-(2,3-dioxoindolin-1-yl)-3-(diphenoxyphosphanyl)succinate (**17**)*

Orange powder; Yield (0.47 g, 90%), mp: 112-114 °C; IR (KBr, υ_{max}): 1731 (C=O), 1615 cm^{-1}.[42]

*Dimethyl 2-[3-(1-cyano-2-ethoxy-2-oxoethylidene)-2-oxoindolin-1-yl]-3-(diphenoxyphosphanyl)succinate (**18**)*

Dark red powder; Yield (0.48 g, 78%), mp: 89-91 °C; IR (KBr, υ_{max}): 2216 (C≡N), 1745, 1726, 1615 (C=O) cm^{-1}; MS (*m/z*, %): 619.5 (M^{+}+1, 5), 618.5 (M^{+}, 2), 573.4 (2), 525.3 (35), 430.2 (58), 241 (10), 223.0 (61), 76.9 (100). Anal. Calcd for $C_{31}H_{27}N_2O_{10}P$ (618.5): C, 60.20; H, 4.40; N, 4.53%. Found: C, 60.28; H, 4.34; N, 4.61%. ^{1}H NMR (250 MHz, $CDCl_3$): δ 1.44 (3H, t, *J*= 7.0 Hz, OCH_2CH_3), 3.75 (3H, s, OCH_3), 3.83-4.30 (1H, m, PC*H*CH), 3.90 (3H, s, OCH_3), 4.46 (2H, q, *J*= 7.0 Hz, OCH_2CH_3), 5.61 (1H, brs, PCHC*H*), 6.90-7.44 (10H, m, 2 OC_6H_5), 7.63 (1H, t, *J*= 8.0 Hz, Ar*H*), 8.30 (1H, d, *J*= 7.5 Hz, Ar*H*), 8.52 (1H, brs, Ar*H*), 8.63 (1H, brs, Ar*H*); ^{13}C NMR (63.0 MHz, $CDCl_3$): δ 10.8 (OCH_2CH_3), 46.0 (d, $^{1}J_{PC}$= 118.0 Hz, P*C*HCH), 45.6 (PCH*C*H), 50.27 and 52.03 (2s, 2 OCH_3), 60.24 (OCH_2CH_3), 107.9 (CH_{Ar}), 116.03 (CN), 109.5 (CH_{Ar}), 110.9 (C=*C*CO_2), 116.9 (C_{Ar}), 120.0 and 120.8 (2s, $4C_{ortho}$), 126.6 and 126.9 (2s, $2C_{para}$), 146.3 (d, $^{2}J_{PC}$=15.1 Hz, $2C_{ipso}$), 132.8 and 135.5 ($4C_{meta}$), 128.4 (*C*=CCO_2), 132.8 (CH_{Ar}), 135.5 (CH_{Ar}), 146.2 (C_{Ar}), 156.4 (N*C*O), 161.5 (CO_2Et), 156.4 ($COCH_3$), 162.7 (P=C-*C*O); ^{31}P NMR (101.2 MHz, $CDCl_3$): δ 10.04 ($O=P(OPh)_2$).

3- ^{1}H, ^{13}C and ^{31}P NMR Spectra of

Compound **4**

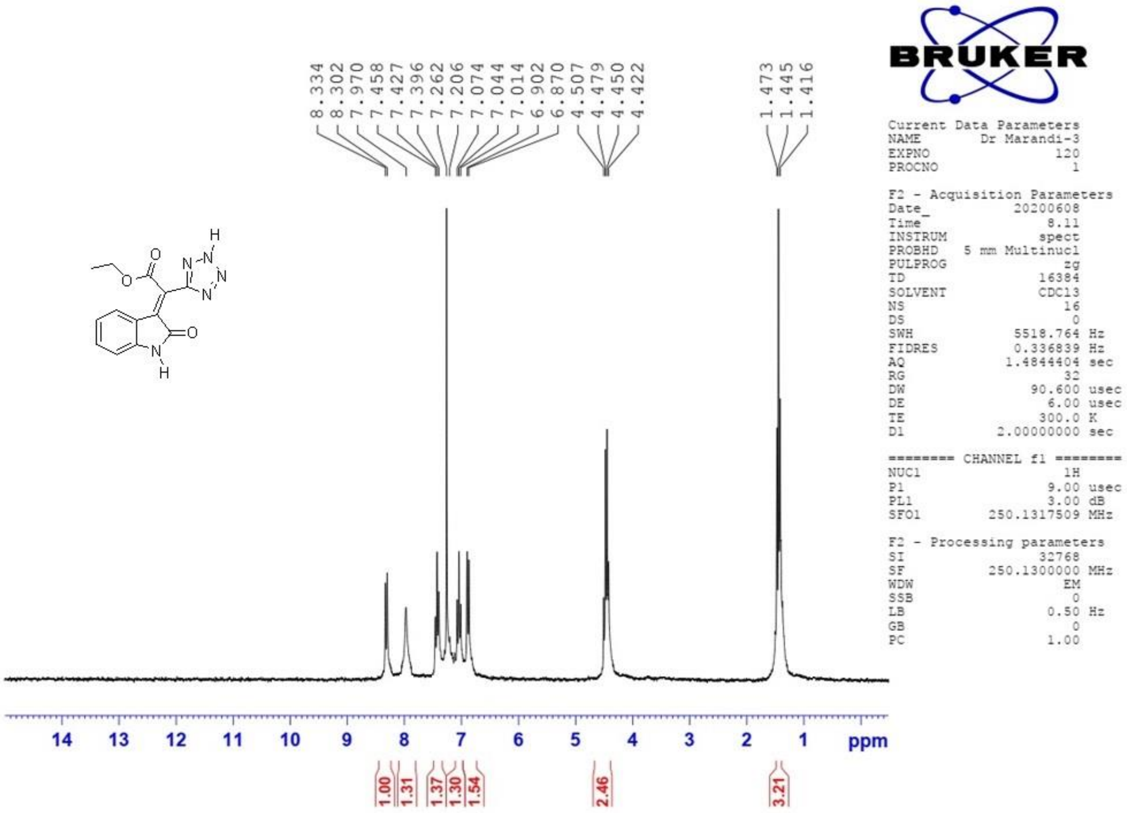

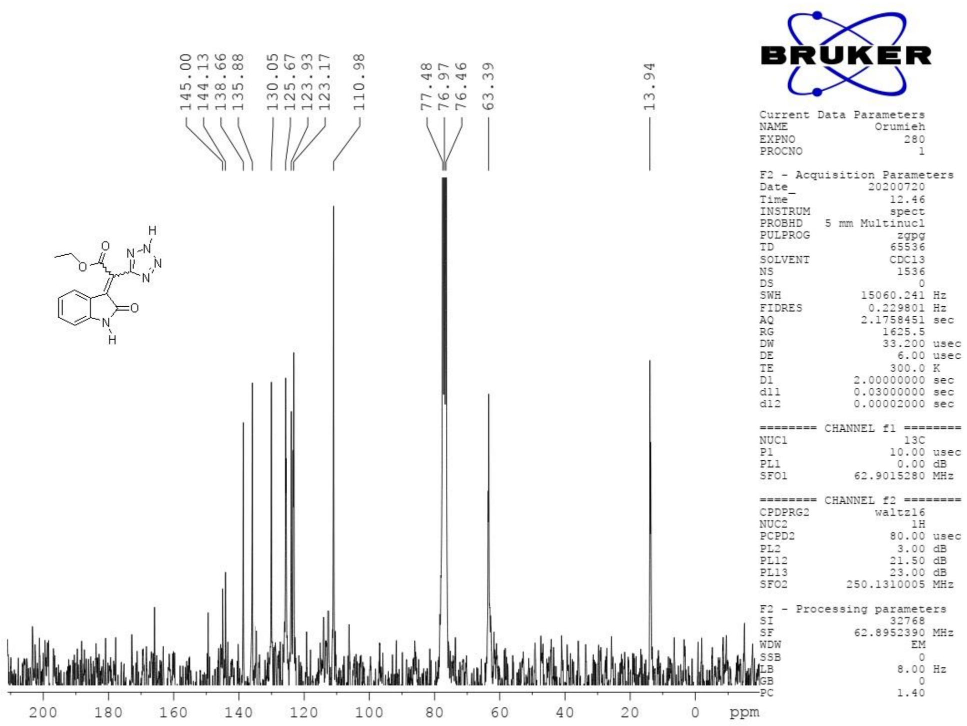
145.00
144.13
138.66
135.88
130.05
125.67
123.93
123.17
110.98
77.48
76.97
76.46
63.39
13.94
BRUKER
Current Data Parameters
NAME Orumieh
EXPNO 280
PROCNO 1
F2 - Acquisition Parameters
Date_ 20200720
Time 12.46
INSTRUM spect
PROBHD 5 mm Multinucl
PULPROG zgpg
TD 65536
SOLVENT CDCl3
NS 1536
DS 0
SWH 15060.241 Hz
FIDRES 0.229801 Hz
AQ 2.1758451 sec
RG 1625.5
DW 33.200 usec
DE 6.00 usec
TE 300.0 K
D1 2.00000000 sec
d11 0.03000000 sec
d12 0.00002000 sec
======== CHANNEL f1 ========
NUC1 13C
P1 10.00 usec
PL1 0.00 dB
SFO1 62.9015280 MHz
======== CHANNEL f2 ========
CPDPRG2 waltz16
NUC2 1H
PCPD2 80.00 usec
PL2 3.00 dB
PL12 21.50 dB
PL13 23.00 dB
SFO2 250.1310005 MHz
F2 - Processing parameters
SI 32768
SF 62.8952390 MHz
WDW EM
SSB 0
LB 8.00 Hz
GB 0
PC 1.40
200 180 160 140 120 100 80 60 40 20 0 ppm

Compound **6**

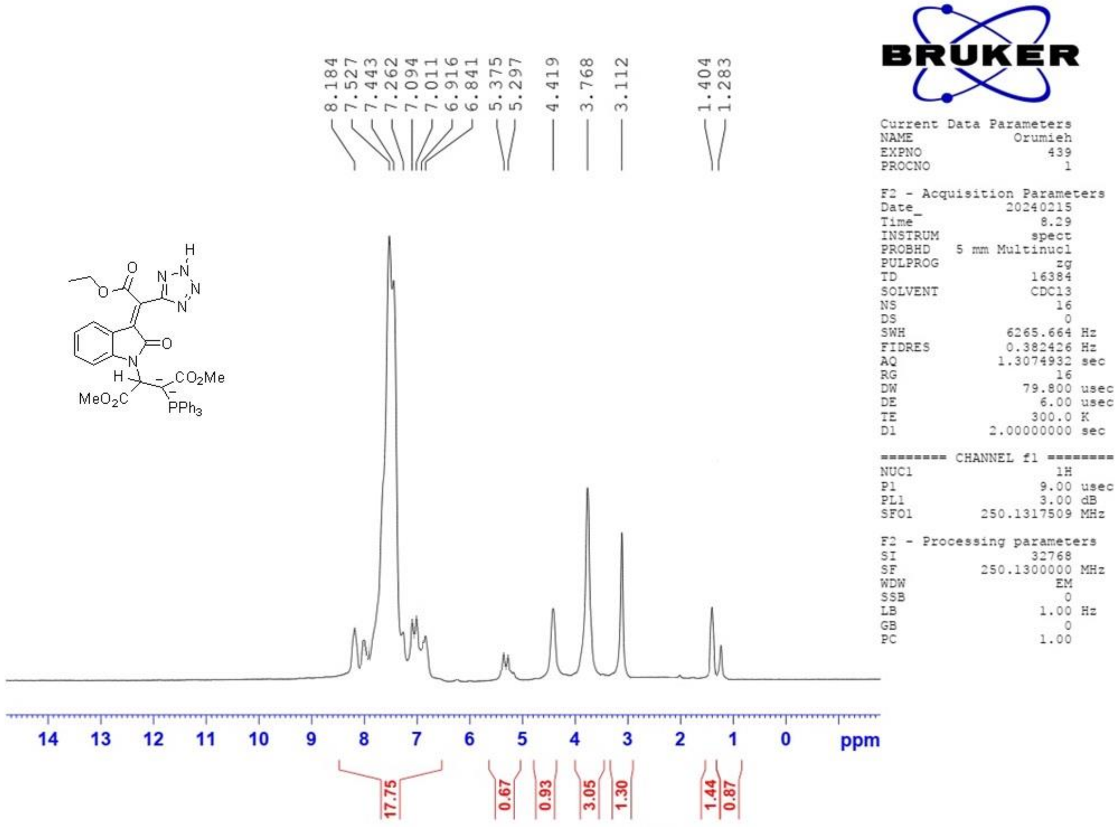
8.184
7.527
7.443
7.262
7.094
7.011
6.916
6.841
5.375
5.297
4.419
3.768
3.112
1.404
1.283
BRUKER
Current Data Parameters
NAME Orumieh
EXPNO 439
PROCNO 1
F2 - Acquisition Parameters
Date_ 20240215
Time 8.29
INSTRUM spect
PROBHD 5 mm Multinucl
PULPROG zg
TD 16384
SOLVENT CDCl3
NS 16
DS 0
SWH 6265.664 Hz
FIDRES 0.382426 Hz
AQ 1.3074932 sec
RG 16
DW 79.800 usec
DE 6.00 usec
TE 300.0 K
D1 2.00000000 sec
======== CHANNEL f1 ========
NUC1 1H
P1 9.00 usec
PL1 3.00 dB
SFO1 250.1317509 MHz
F2 - Processing parameters
SI 32768
SF 250.1300000 MHz
WDW EM
SSB 0
LB 1.00 Hz
GB 0
PC 1.00
14 13 12 11 10 9 8 7 6 5 4 3 2 1 0 ppm
17.75
0.67
0.93
3.05
1.30
1.44
0.87

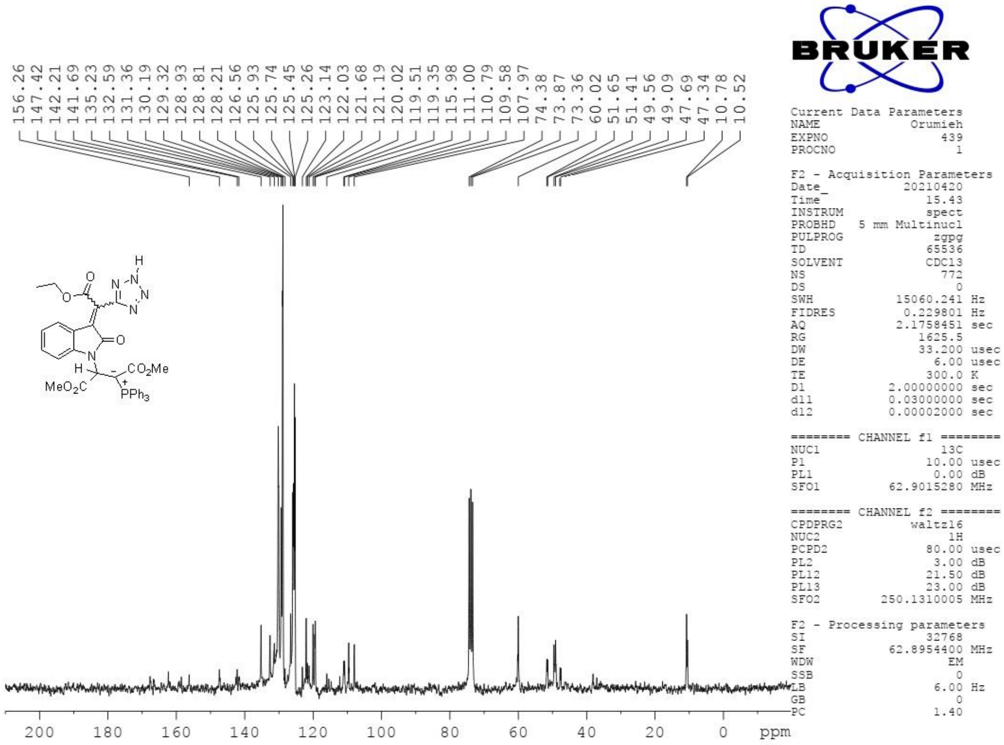

156.26 147.42 142.21 141.69 135.23 132.59 131.36 130.19 129.32 128.93 128.81 128.21 126.56 125.93 125.74 125.45 125.26 123.14 122.03 121.68 121.19 120.02 119.51 119.35 115.98 111.00 110.79 109.58 107.97 74.38 73.87 73.36 60.02 51.65 51.41 49.56 49.09 47.69 47.34 10.78 10.52
BRUKER
Current Data Parameters
NAME Orumieh
EXPNO 439
PROCNO 1
F2 - Acquisition Parameters
Date_ 20210420
Time 15.43
INSTRUM spect
PROBHD 5 mm Multinucl
PULPROG zgpg
TD 65536
SOLVENT CDCl3
NS 772
DS 0
SWH 15060.241 Hz
FIDRES 0.229801 Hz
AQ 2.1758451 sec
RG 1625.5
DW 33.200 usec
DE 6.00 usec
TE 300.0 K
D1 2.00000000 sec
d11 0.03000000 sec
d12 0.00002000 sec
======== CHANNEL f1 ========
NUC1 13C
P1 10.00 usec
PL1 0.00 dB
SFO1 62.9015280 MHz
======== CHANNEL f2 ========
CPDPRG2 waltz16
NUC2 1H
PCPD2 80.00 usec
PL2 3.00 dB
PL12 21.50 dB
PL13 23.00 dB
SFO2 250.1310005 MHz
F2 - Processing parameters
SI 32768
SF 62.8954400 MHz
WDW EM
SSB 0
LB 6.00 Hz
GB 0
PC 1.40
200 180 160 140 120 100 80 60 40 20 0 ppm

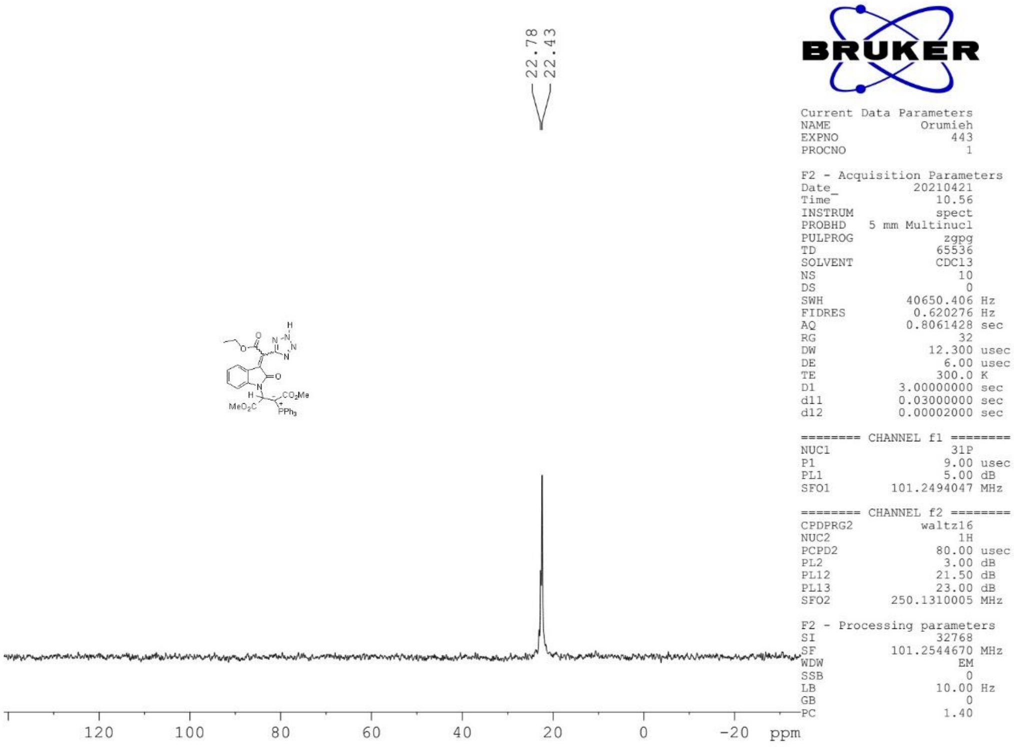

22.78 22.43
BRUKER
Current Data Parameters
NAME Orumieh
EXPNO 443
PROCNO 1
F2 - Acquisition Parameters
Date_ 20210421
Time 10.56
INSTRUM spect
PROBHD 5 mm Multinucl
PULPROG zgpg
TD 65536
SOLVENT CDCl3
NS 10
DS 0
SWH 40650.406 Hz
FIDRES 0.620276 Hz
AQ 0.8061428 sec
RG 32
DW 12.300 usec
DE 6.00 usec
TE 300.0 K
D1 3.00000000 sec
d11 0.03000000 sec
d12 0.00002000 sec
======== CHANNEL f1 ========
NUC1 31P
P1 9.00 usec
PL1 5.00 dB
SFO1 101.2494047 MHz
======== CHANNEL f2 ========
CPDPRG2 waltz16
NUC2 1H
PCPD2 80.00 usec
PL2 3.00 dB
PL12 21.50 dB
PL13 23.00 dB
SFO2 250.1310005 MHz
F2 - Processing parameters
SI 32768
SF 101.2544670 MHz
WDW EM
SSB 0
LB 10.00 Hz
GB 0
PC 1.40
120 100 80 60 40 20 0 -20 ppm

Compound **10**

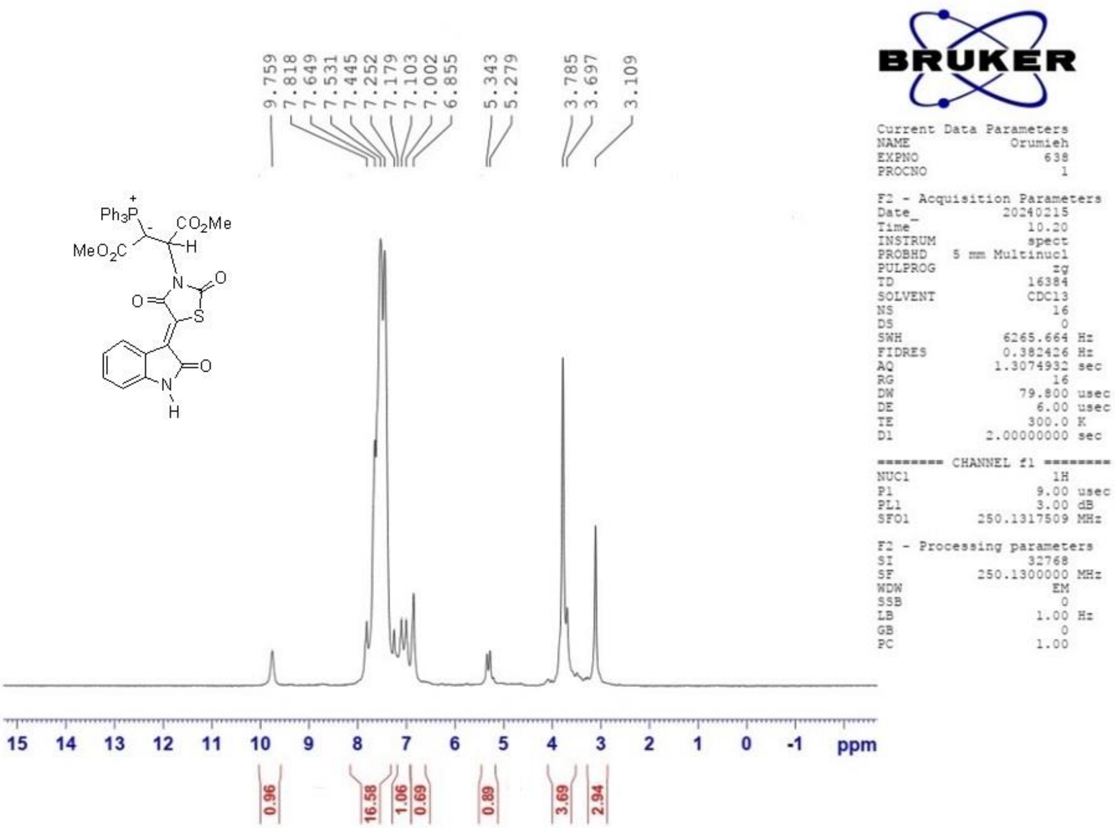
9.759
7.818
7.649
7.531
7.445
7.252
7.179
7.103
7.002
6.855
5.343
5.279
3.785
3.697
3.109
BRUKER
Current Data Parameters
NAME Orumieh
EXPNO 638
PROCNO 1
F2 - Acquisition Parameters
Date_ 20240215
Time 10.20
INSTRUM spect
PROBHD 5 mm Multinucl
PULPROG zg
TD 16384
SOLVENT CDCl3
NS 16
DS 0
SWH 6265.664 Hz
FIDRES 0.382426 Hz
AQ 1.3074932 sec
RG 16
DW 79.800 usec
DE 6.00 usec
TE 300.0 K
D1 2.00000000 sec
======== CHANNEL f1 ========
NUC1 1H
P1 9.00 usec
PL1 3.00 dB
SFO1 250.1317509 MHz
F2 - Processing parameters
SI 32768
SF 250.1300000 MHz
WDW EM
SSB 0
LB 1.00 Hz
GB 0
PC 1.00
15 14 13 12 11 10 9 8 7 6 5 4 3 2 1 0 -1 ppm
0.96
16.58
1.06
0.69
0.89
3.69
2.94

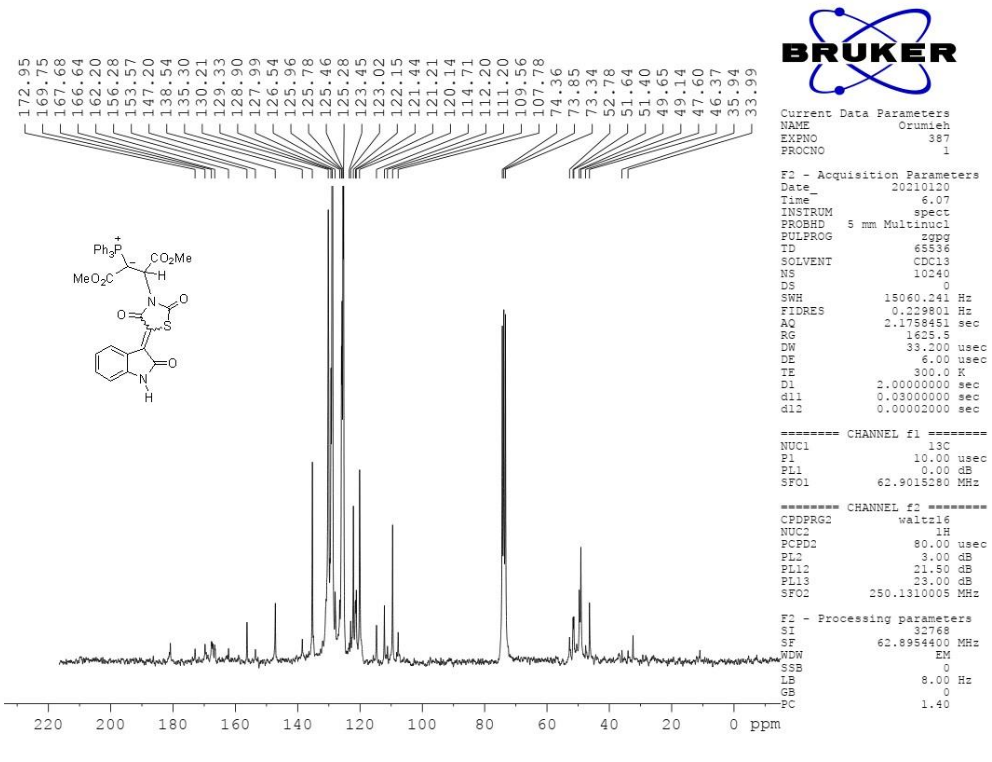
172.95
169.75
167.68
166.64
162.20
156.28
153.57
147.20
138.54
135.30
130.21
129.33
128.90
127.99
126.54
125.96
125.78
125.46
125.28
123.45
123.02
122.15
121.44
121.21
120.14
114.71
112.20
111.20
109.56
107.78
74.36
73.85
73.34
52.78
51.64
51.40
49.65
49.14
47.60
46.37
35.94
33.99
BRUKER
Current Data Parameters
NAME Orumieh
EXPNO 387
PROCNO 1
F2 - Acquisition Parameters
Date_ 20210120
Time 6.07
INSTRUM spect
PROBHD 5 mm Multinucl
PULPROG zgpg
TD 65536
SOLVENT CDCl3
NS 10240
DS 0
SWH 15060.241 Hz
FIDRES 0.229801 Hz
AQ 2.1758451 sec
RG 1625.5
DW 33.200 usec
DE 6.00 usec
TE 300.0 K
D1 2.00000000 sec
d11 0.03000000 sec
d12 0.00002000 sec
======== CHANNEL f1 ========
NUC1 13C
P1 10.00 usec
PL1 0.00 dB
SFO1 62.9015280 MHz
======== CHANNEL f2 ========
CPDPRG2 waltz16
NUC2 1H
PCPD2 80.00 usec
PL2 3.00 dB
PL12 21.50 dB
PL13 23.00 dB
SFO2 250.1310005 MHz
F2 - Processing parameters
SI 32768
SF 62.8954400 MHz
WDW EM
SSB 0
LB 8.00 Hz
GB 0
PC 1.40
220 200 180 160 140 120 100 80 60 40 20 0 ppm

11

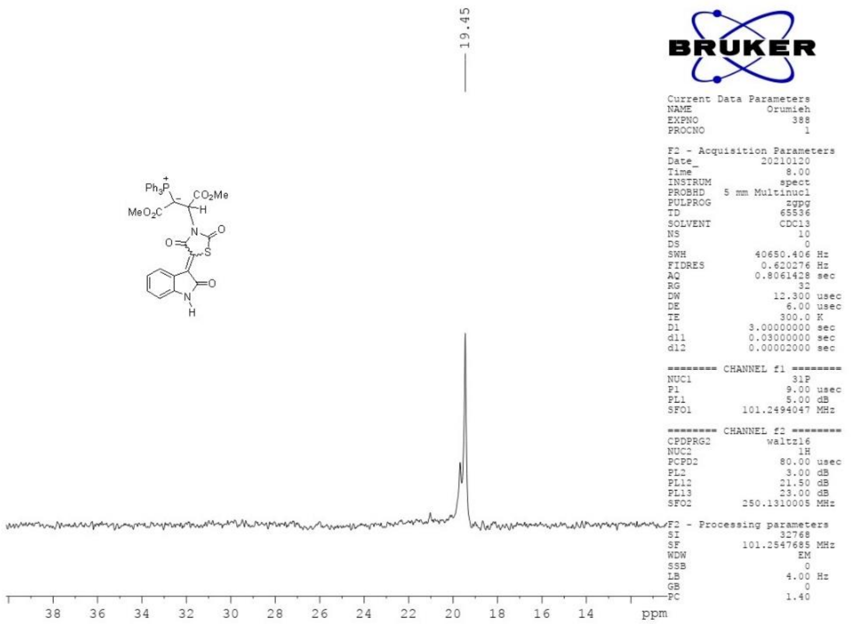

Compound **11**

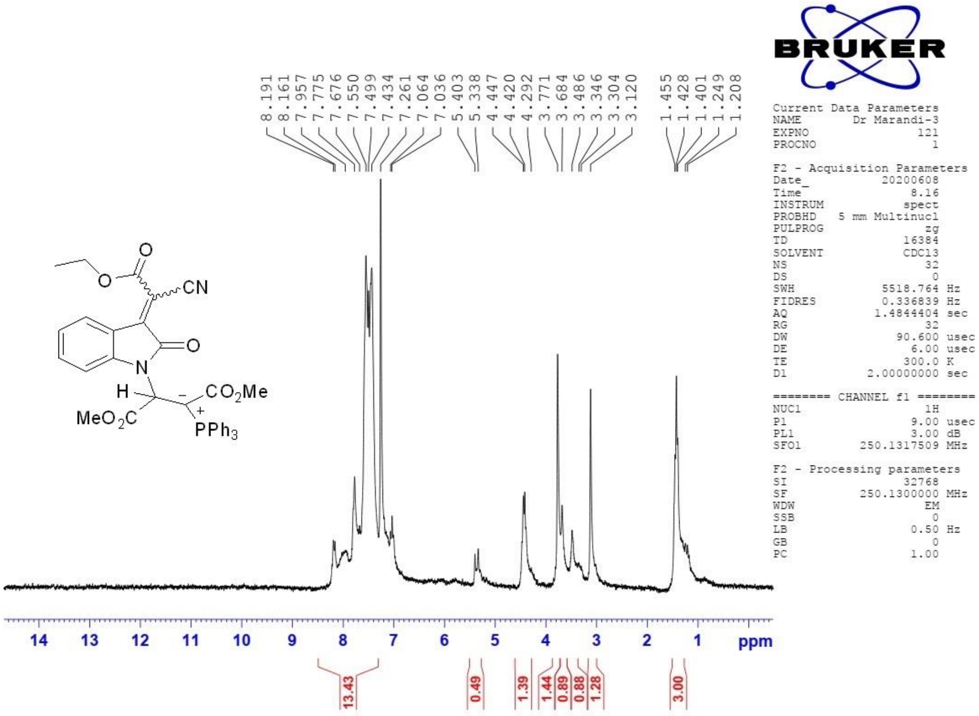

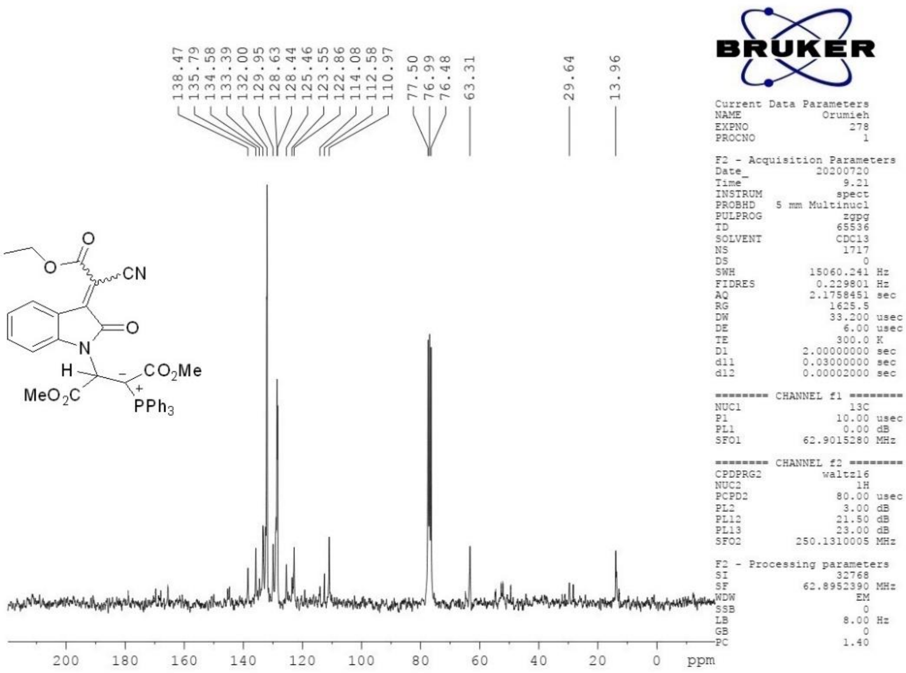
138.47
135.79
134.58
133.39
132.00
129.95
128.63
128.44
125.46
123.55
122.86
114.08
112.58
110.97
77.50
76.99
76.48
63.31
29.64
13.96
BRUKER
Current Data Parameters
NAME Orumieh
EXPNO 278
PROCNO 1
F2 - Acquisition Parameters
Date_ 20200720
Time 9.21
INSTRUM spect
PROBHD 5 mm Multinucl
PULPROG zgpg
TD 65536
SOLVENT CDCl3
NS 1717
DS 0
SWH 15060.241 Hz
FIDRES 0.229801 Hz
AQ 2.1758451 sec
RG 1625.5
DW 33.200 usec
DE 6.00 usec
TE 300.0 K
D1 2.00000000 sec
d11 0.03000000 sec
d12 0.00002000 sec
======== CHANNEL f1 ========
NUC1 13C
P1 10.00 usec
PL1 0.00 dB
SFO1 62.9015280 MHz
======== CHANNEL f2 ========
CPDPRG2 waltz16
NUC2 1H
PCPD2 80.00 usec
PL2 3.00 dB
PL12 21.50 dB
PL13 23.00 dB
SFO2 250.1310005 MHz
F2 - Processing parameters
SI 32768
SF 62.8952390 MHz
WDW EM
SSB 0
LB 8.00 Hz
GB 0
PC 1.40
CN
O
N
H
CO2Me
MeO2C
PPh3
200 180 160 140 120 100 80 60 40 20 0 ppm

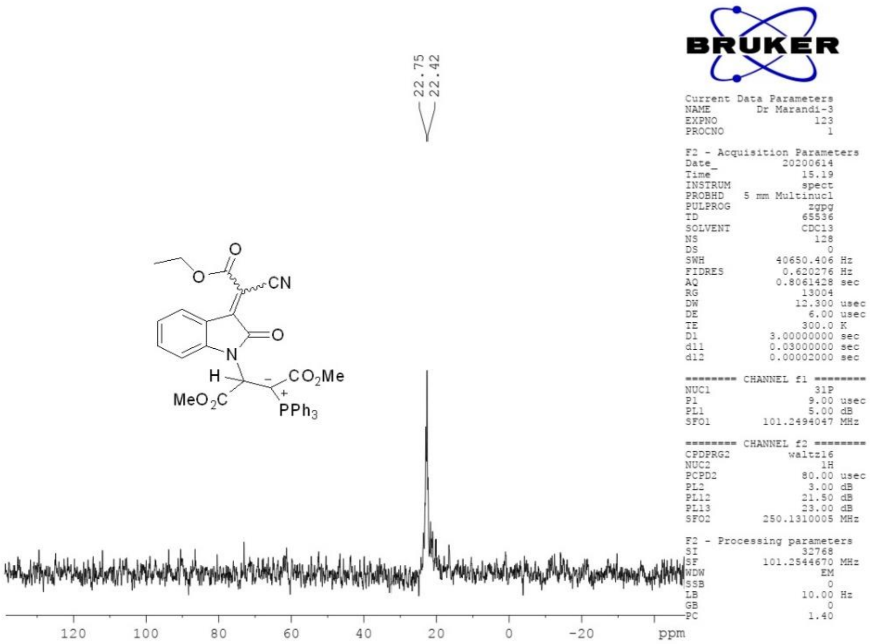
22.75
22.42
BRUKER
Current Data Parameters
NAME Dr Marandi-3
EXPNO 123
PROCNO 1
F2 - Acquisition Parameters
Date_ 20200614
Time 15.19
INSTRUM spect
PROBHD 5 mm Multinucl
PULPROG zgpg
TD 65536
SOLVENT CDCl3
NS 128
DS 0
SWH 40650.406 Hz
FIDRES 0.620276 Hz
AQ 0.8061428 sec
RG 13004
DW 12.300 usec
DE 6.00 usec
TE 300.0 K
D1 3.00000000 sec
d11 0.03000000 sec
d12 0.00002000 sec
======== CHANNEL f1 ========
NUC1 31P
P1 9.00 usec
PL1 5.00 dB
SFO1 101.2494047 MHz
======== CHANNEL f2 ========
CPDPRG2 waltz16
NUC2 1H
PCPD2 80.00 usec
PL2 3.00 dB
PL12 21.50 dB
PL13 23.00 dB
SFO2 250.1310005 MHz
F2 - Processing parameters
SI 32768
SF 101.2544670 MHz
WDW EM
SSB 0
LB 10.00 Hz
GB 0
PC 1.40
CN
O
N
H
CO2Me
MeO2C
PPh3
120 100 80 60 40 20 0 -20 ppm

Compound **12**

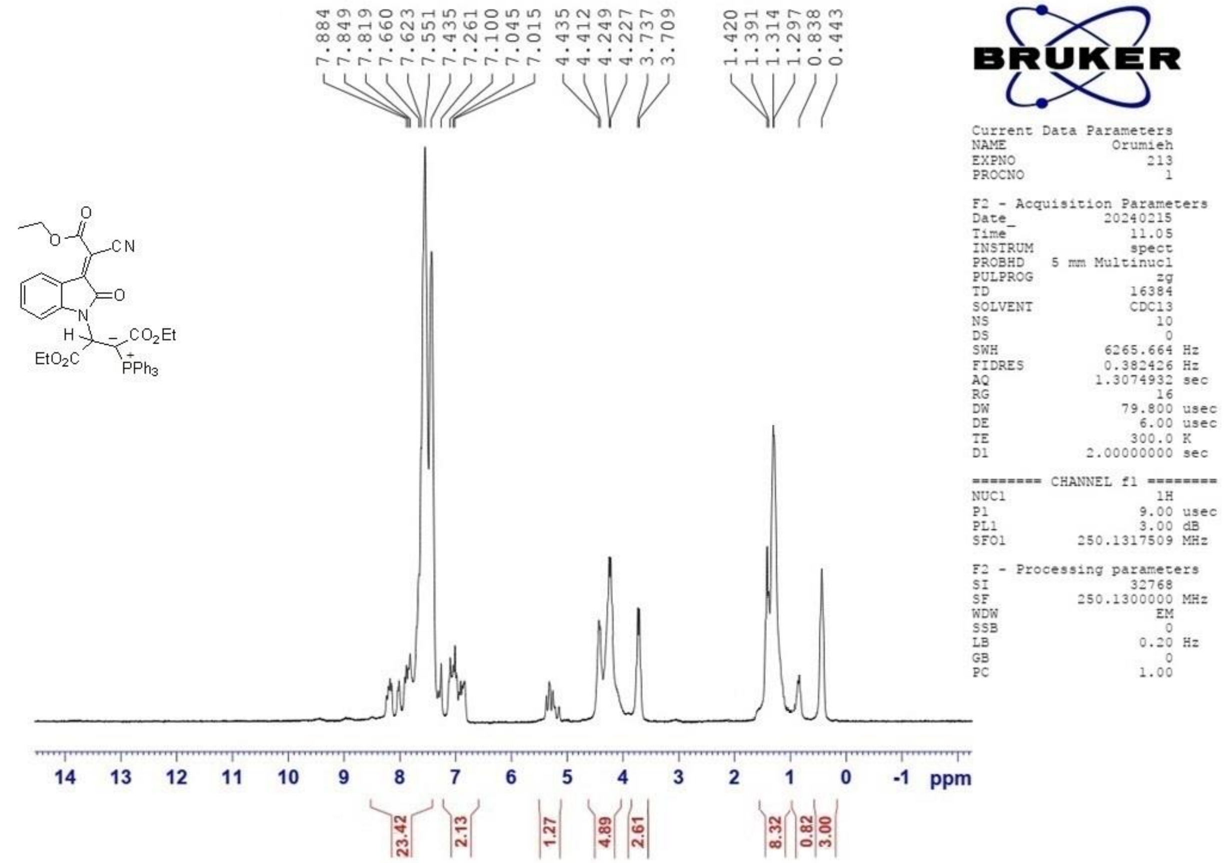
7.884
7.849
7.819
7.660
7.623
7.551
7.435
7.261
7.100
7.045
7.015
4.435
4.412
4.249
4.227
3.737
3.709
1.420
1.391
1.314
1.297
0.838
0.443
CN
O
N
H
CO2Et
EtO2C
PPh3
BRUKER
Current Data Parameters
NAME Orumieh
EXPNO 213
PROCNO 1
F2 - Acquisition Parameters
Date_ 20240215
Time 11.05
INSTRUM spect
PROBHD 5 mm Multinucl
PULPROG zg
TD 16384
SOLVENT CDCl3
NS 10
DS 0
SWH 6265.664 Hz
FIDRES 0.382426 Hz
AQ 1.3074932 sec
RG 16
DW 79.800 usec
DE 6.00 usec
TE 300.0 K
D1 2.00000000 sec
======== CHANNEL f1 ========
NUC1 1H
P1 9.00 usec
PL1 3.00 dB
SFO1 250.1317509 MHz
F2 - Processing parameters
SI 32768
SF 250.1300000 MHz
WDW EM
SSB 0
LB 0.20 Hz
GB 0
PC 1.00
14 13 12 11 10 9 8 7 6 5 4 3 2 1 0 -1 ppm
23.42
2.13
1.27
4.89
2.61
8.32
0.82
3.00

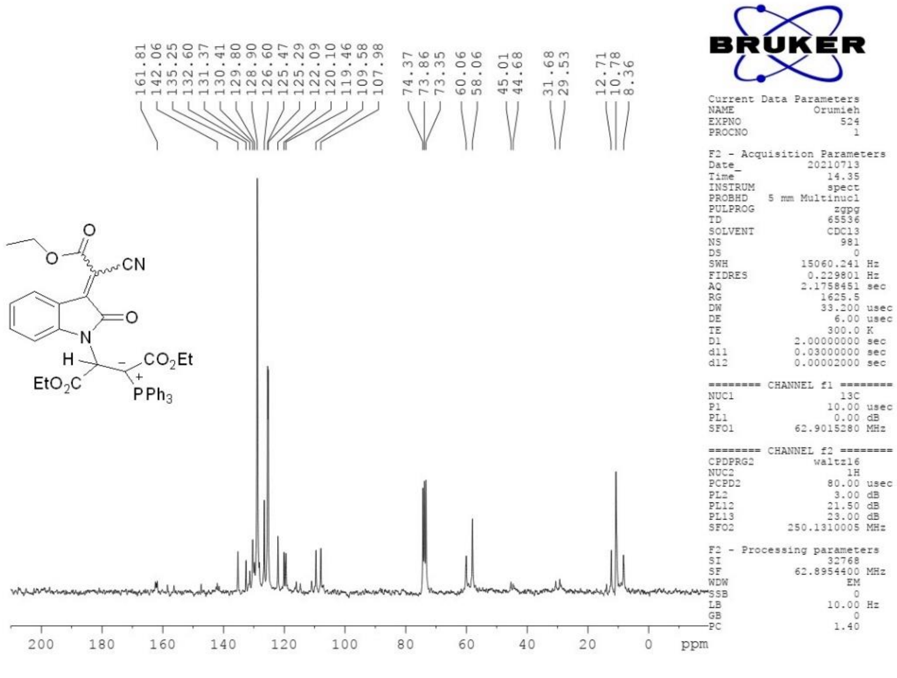
161.81
142.06
135.25
132.60
131.37
130.41
129.80
128.90
126.60
125.47
125.29
122.09
120.10
119.46
109.58
107.98
74.37
73.86
73.35
60.06
58.06
45.01
44.68
31.68
29.53
12.71
10.78
8.36
CN
O
N
H
CO2Et
EtO2C
PPh3
BRUKER
Current Data Parameters
NAME Orumieh
EXPNO 524
PROCNO 1
F2 - Acquisition Parameters
Date_ 20210713
Time 14.35
INSTRUM spect
PROBHD 5 mm Multinucl
PULPROG zgpg
TD 65536
SOLVENT CDCl3
NS 981
DS 0
SWH 15060.241 Hz
FIDRES 0.229801 Hz
AQ 2.1758451 sec
RG 1625.5
DW 33.200 usec
DE 6.00 usec
TE 300.0 K
D1 2.00000000 sec
d11 0.03000000 sec
d12 0.00002000 sec
======== CHANNEL f1 ========
NUC1 13C
P1 10.00 usec
PL1 0.00 dB
SFO1 62.9015280 MHz
======== CHANNEL f2 ========
CPDPRG2 waltz16
NUC2 1H
PCPD2 80.00 usec
PL2 3.00 dB
PL12 21.50 dB
PL13 23.00 dB
SFO2 250.1310005 MHz
F2 - Processing parameters
SI 32768
SF 62.8954400 MHz
WDW EM
SSB 0
LB 10.00 Hz
GB 0
PC 1.40
200 180 160 140 120 100 80 60 40 20 0 ppm

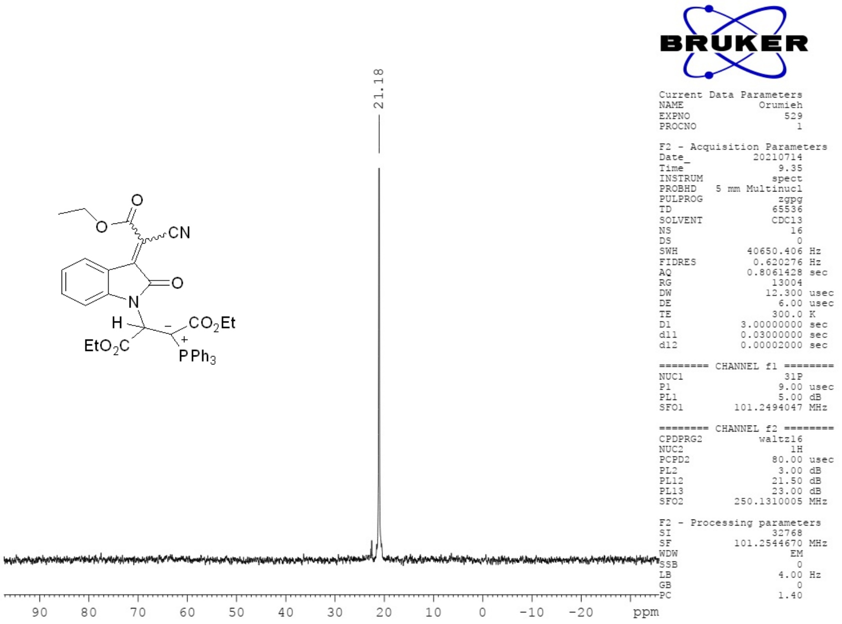

21.18
CN
O
N
H
CO_2Et
EtO_2C
PPh_3
BRUKER
Current Data Parameters
NAME Orumieh
EXPNO 529
PROCNO 1
F2 - Acquisition Parameters
Date_ 20210714
Time 9.35
INSTRUM spect
PROBHD 5 mm Multinucl
PULPROG zgpg
TD 65536
SOLVENT CDCl3
NS 16
DS 0
SWH 40650.406 Hz
FIDRES 0.620276 Hz
AQ 0.8061428 sec
RG 13004
DW 12.300 usec
DE 6.00 usec
TE 300.0 K
D1 3.00000000 sec
d11 0.03000000 sec
d12 0.00002000 sec
======== CHANNEL f1 ========
NUC1 31P
P1 9.00 usec
PL1 5.00 dB
SFO1 101.2494047 MHz
======== CHANNEL f2 ========
CPDPRG2 waltz16
NUC2 1H
PCPD2 80.00 usec
PL2 3.00 dB
PL12 21.50 dB
PL13 23.00 dB
SFO2 250.1310005 MHz
F2 - Processing parameters
SI 32768
SF 101.2544670 MHz
WDW EM
SSB 0
LB 4.00 Hz
GB 0
PC 1.40
90 80 70 60 50 40 30 20 10 0 -10 -20 ppm

Compound **13**

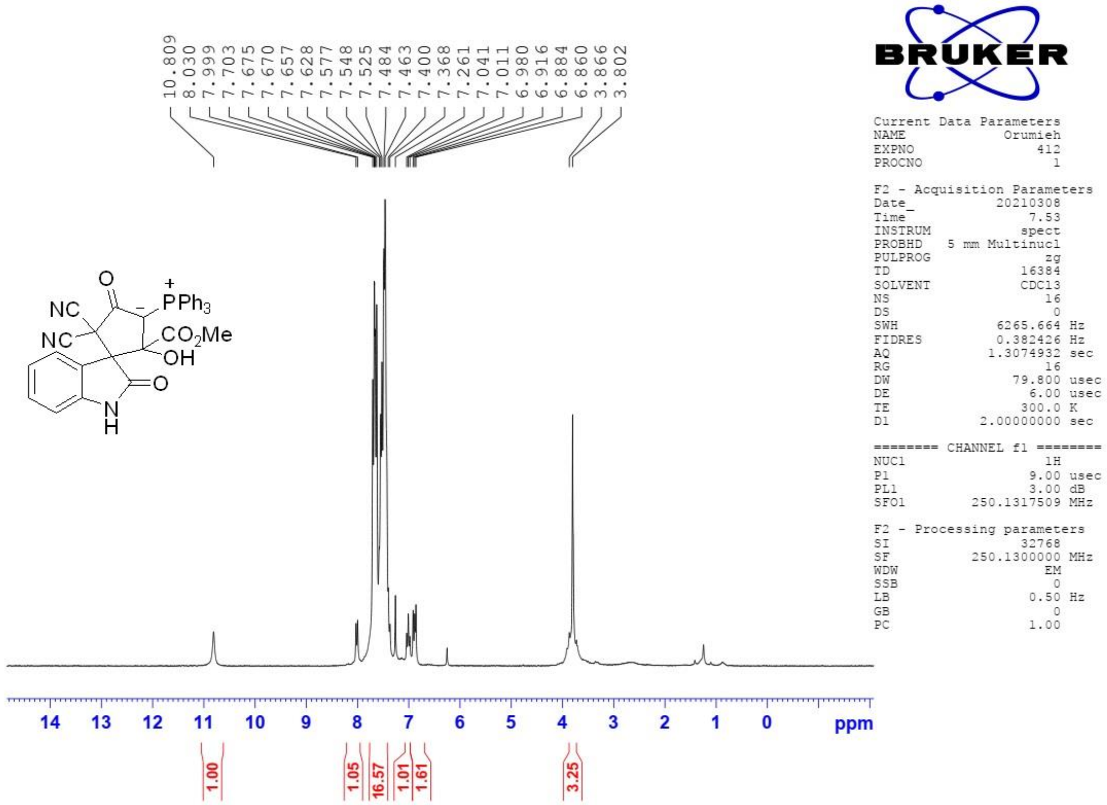

10.809 8.030 7.999 7.703 7.675 7.670 7.657 7.628 7.577 7.548 7.525 7.484 7.463 7.400 7.368 7.261 7.041 7.011 6.980 6.916 6.884 6.860 3.866 3.802
NC
NC
O
PPh_3
CO_2Me
OH
O
N
H
BRUKER
Current Data Parameters
NAME Orumieh
EXPNO 412
PROCNO 1
F2 - Acquisition Parameters
Date_ 20210308
Time 7.53
INSTRUM spect
PROBHD 5 mm Multinucl
PULPROG zg
TD 16384
SOLVENT CDCl3
NS 16
DS 0
SWH 6265.664 Hz
FIDRES 0.382426 Hz
AQ 1.3074932 sec
RG 16
DW 79.800 usec
DE 6.00 usec
TE 300.0 K
D1 2.00000000 sec
======== CHANNEL f1 ========
NUC1 1H
P1 9.00 usec
PL1 3.00 dB
SFO1 250.1317509 MHz
F2 - Processing parameters
SI 32768
SF 250.1300000 MHz
WDW EM
SSB 0
LB 0.50 Hz
GB 0
PC 1.00
14 13 12 11 10 9 8 7 6 5 4 3 2 1 0 ppm
1.00 1.05 16.57 1.01 1.61 3.25

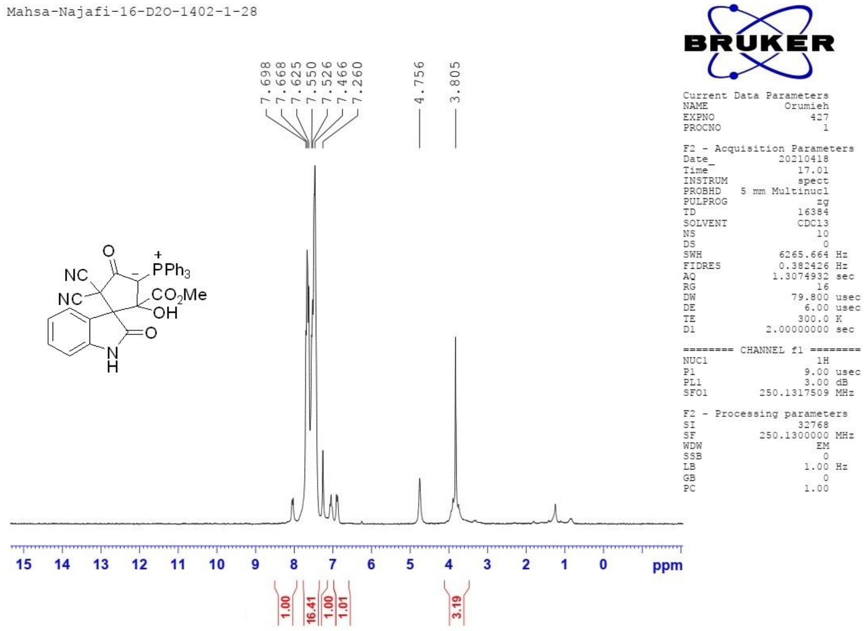
Mahsa-Najafi-16-D2O-1402-1-28
7.698
7.668
7.625
7.550
7.526
7.466
7.260
4.756
3.805
BRUKER
Current Data Parameters
NAME Orumieh
EXPNO 427
PROCNO 1
F2 - Acquisition Parameters
Date_ 20210418
Time 17.01
INSTRUM spect
PROBHD 5 mm Multinucl
PULPROG zg
TD 16384
SOLVENT CDCl3
NS 10
DS 0
SWH 6265.664 Hz
FIDRES 0.382426 Hz
AQ 1.3074932 sec
RG 16
DW 79.800 usec
DE 6.00 usec
TE 300.0 K
D1 2.00000000 sec
======== CHANNEL f1 ========
NUC1 1H
P1 9.00 usec
PL1 3.00 dB
SFO1 250.1317509 MHz
F2 - Processing parameters
SI 32768
SF 250.1300000 MHz
WDW EM
SSB 0
LB 1.00 Hz
GB 0
PC 1.00
15 14 13 12 11 10 9 8 7 6 5 4 3 2 1 0 ppm
1.00
16.41
1.00
1.01
3.19

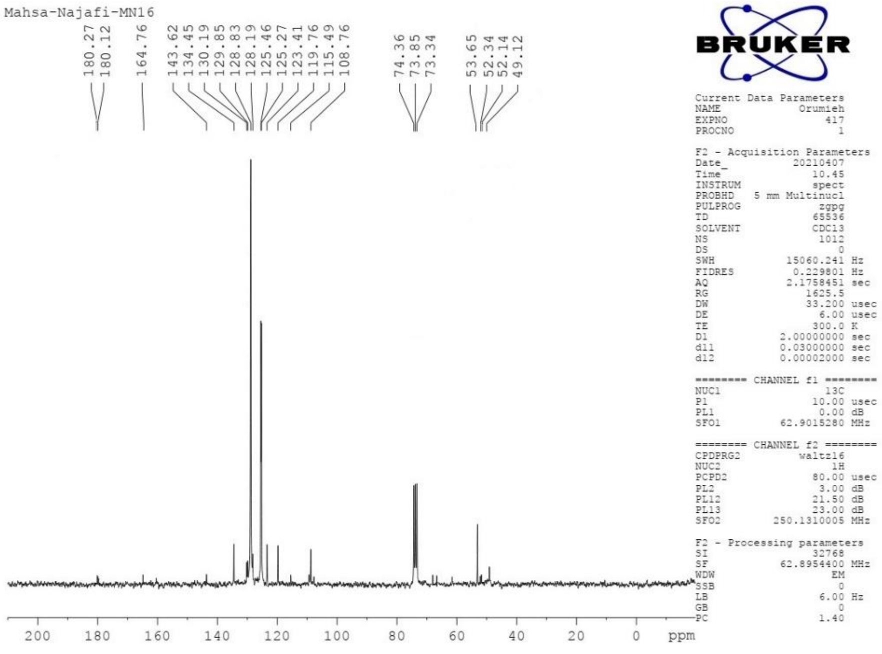
Mahsa-Najafi-MN16
180.27
180.12
164.76
143.62
134.45
130.19
129.85
128.83
128.19
125.46
125.27
123.41
119.76
115.49
108.76
74.36
73.85
73.34
53.65
52.34
52.14
49.12
BRUKER
Current Data Parameters
NAME Orumieh
EXPNO 417
PROCNO 1
F2 - Acquisition Parameters
Date_ 20210407
Time 10.45
INSTRUM spect
PROBHD 5 mm Multinucl
PULPROG zgpg
TD 65536
SOLVENT CDCl3
NS 1012
DS 0
SWH 15060.241 Hz
FIDRES 0.229801 Hz
AQ 2.1758451 sec
RG 1625.5
DW 33.200 usec
DE 6.00 usec
TE 300.0 K
D1 2.00000000 sec
d11 0.03000000 sec
d12 0.00002000 sec
======== CHANNEL f1 ========
NUC1 13C
P1 10.00 usec
PL1 0.00 dB
SFO1 62.9015280 MHz
======== CHANNEL f2 ========
CPDPRG2 waltz16
NUC2 1H
PCPD2 80.00 usec
PL2 3.00 dB
PL12 21.50 dB
PL13 23.00 dB
SFO2 250.1310005 MHz
F2 - Processing parameters
SI 32768
SF 62.8954400 MHz
WDW EM
SSB 0
LB 6.00 Hz
GB 0
PC 1.40
200 180 160 140 120 100 80 60 40 20 0 ppm

16

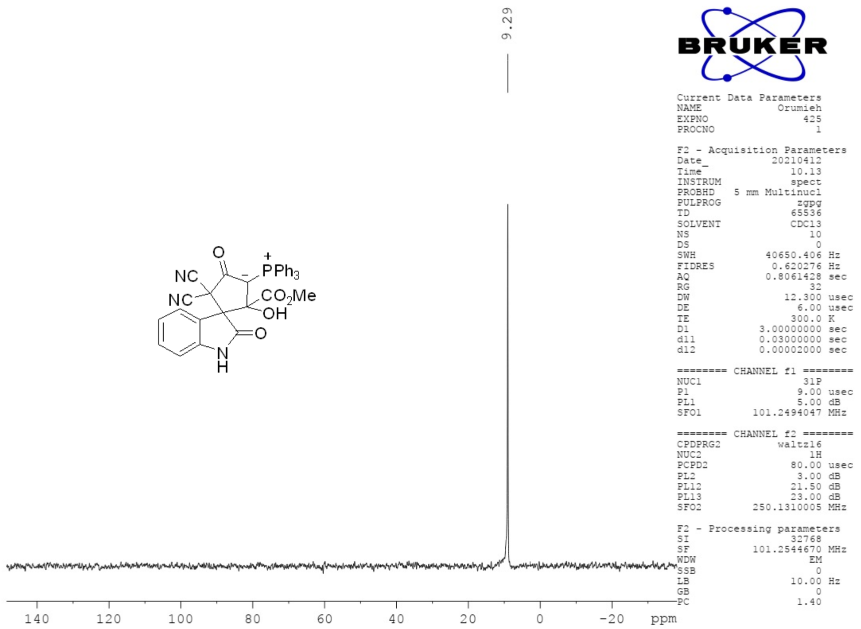

Compound **15**

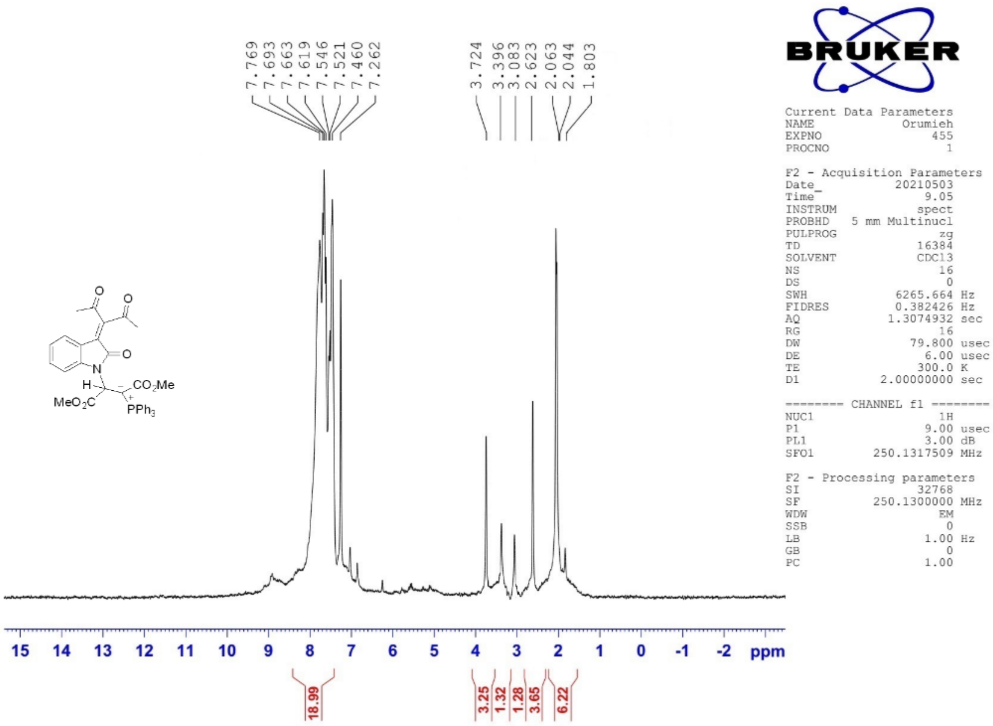

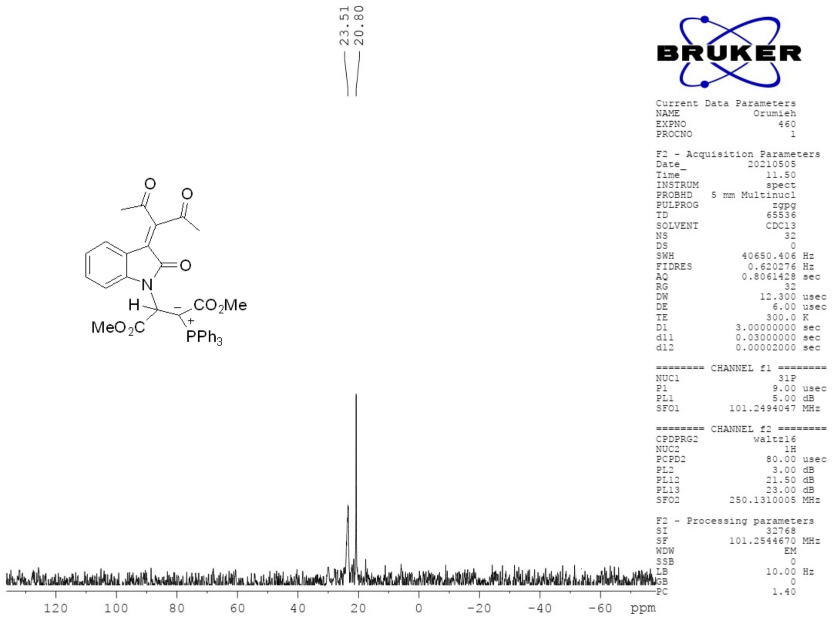

23.51
20.80
BRUKER
Current Data Parameters
NAME Orumieh
EXPNO 460
PROCNO 1
F2 - Acquisition Parameters
Date_ 20210505
Time 11.50
INSTRUM spect
PROBHD 5 mm Multinucl
PULPROG zgpg
TD 65536
SOLVENT CDCl3
NS 32
DS 0
SWH 40650.406 Hz
FIDRES 0.620276 Hz
AQ 0.8061428 sec
RG 32
DW 12.300 usec
DE 6.00 usec
TE 300.0 K
D1 3.00000000 sec
d11 0.03000000 sec
d12 0.00002000 sec
======== CHANNEL f1 ========
NUC1 31P
P1 9.00 usec
PL1 5.00 dB
SFO1 101.2494047 MHz
======== CHANNEL f2 ========
CPDPRG2 waltz16
NUC2 1H
PCPD2 80.00 usec
PL2 3.00 dB
PL12 21.50 dB
PL13 23.00 dB
SFO2 250.1310005 MHz
F2 - Processing parameters
SI 32768
SF 101.2544670 MHz
WDW EM
SSB 0
LB 10.00 Hz
GB 0
PC 1.40
120 100 80 60 40 20 0 -20 -40 -60 ppm

Compound **16**

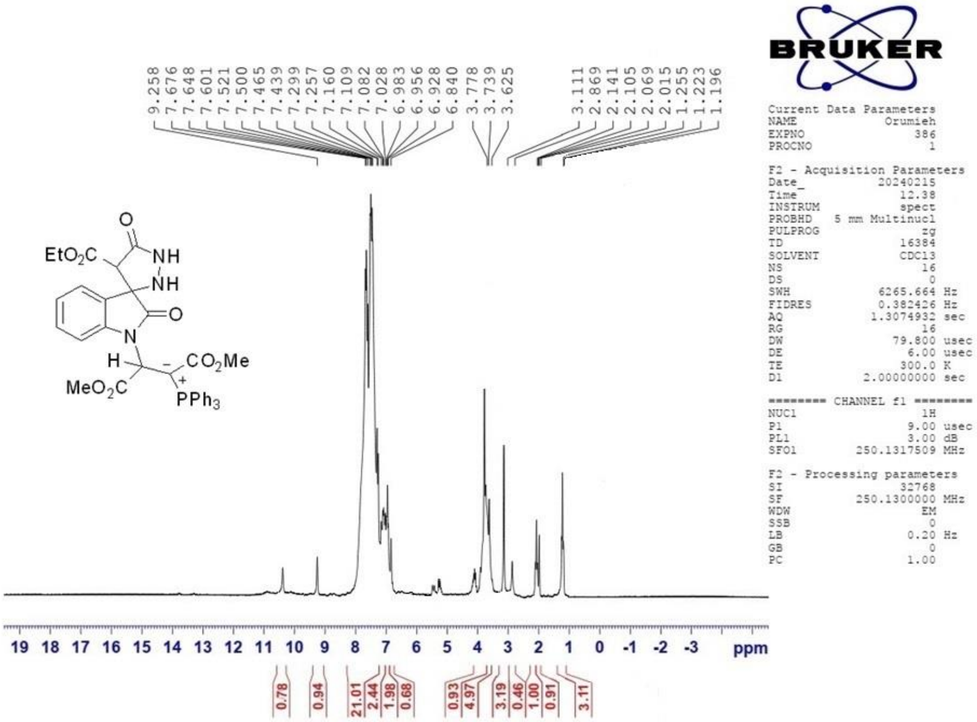

9.258 7.676 7.648 7.601 7.521 7.500 7.465 7.439 7.299 7.257 7.160 7.109 7.082 7.028 6.983 6.956 6.928 6.840 3.778 3.739 3.625 3.111 2.869 2.141 2.105 2.069 2.015 1.255 1.223 1.196
BRUKER
Current Data Parameters
NAME Orumieh
EXPNO 386
PROCNO 1
F2 - Acquisition Parameters
Date_ 20240215
Time 12.38
INSTRUM spect
PROBHD 5 mm Multinucl
PULPROG zg
TD 16384
SOLVENT CDCl3
NS 16
DS 0
SWH 6265.664 Hz
FIDRES 0.382426 Hz
AQ 1.3074932 sec
RG 16
DW 79.800 usec
DE 6.00 usec
TE 300.0 K
D1 2.00000000 sec
======== CHANNEL f1 ========
NUC1 1H
P1 9.00 usec
PL1 3.00 dB
SFO1 250.1317509 MHz
F2 - Processing parameters
SI 32768
SF 250.1300000 MHz
WDW EM
SSB 0
LB 0.20 Hz
GB 0
PC 1.00
19 18 17 16 15 14 13 12 11 10 9 8 7 6 5 4 3 2 1 0 -1 -2 -3 ppm
0.78 0.94 21.01 2.44 1.98 0.68 0.93 4.97 3.19 0.46 1.00 0.91 3.11

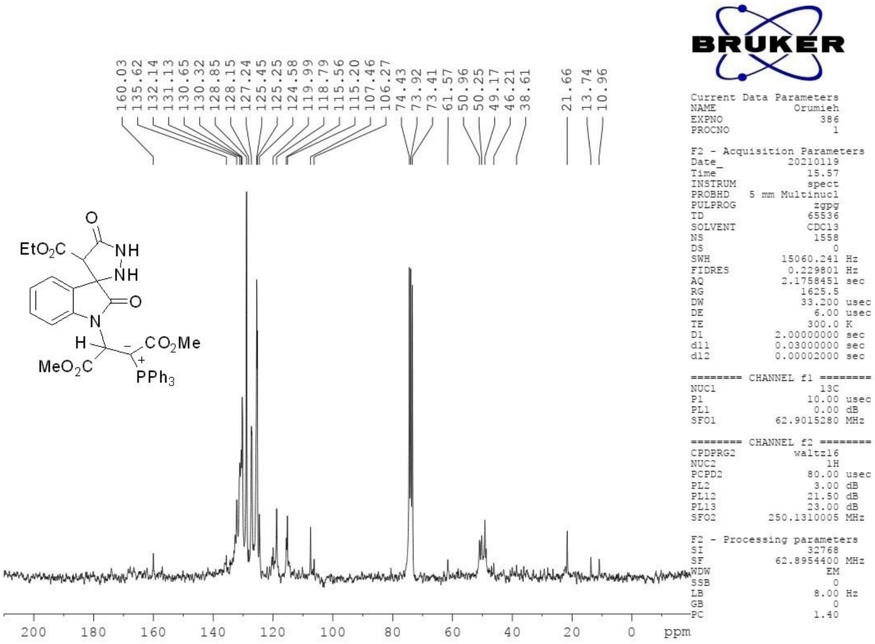

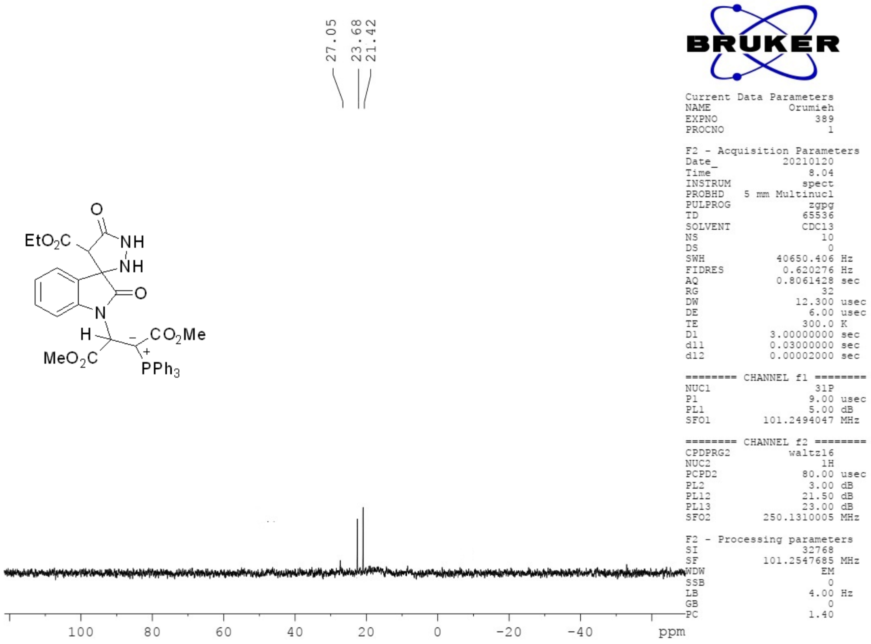

Compound **18**

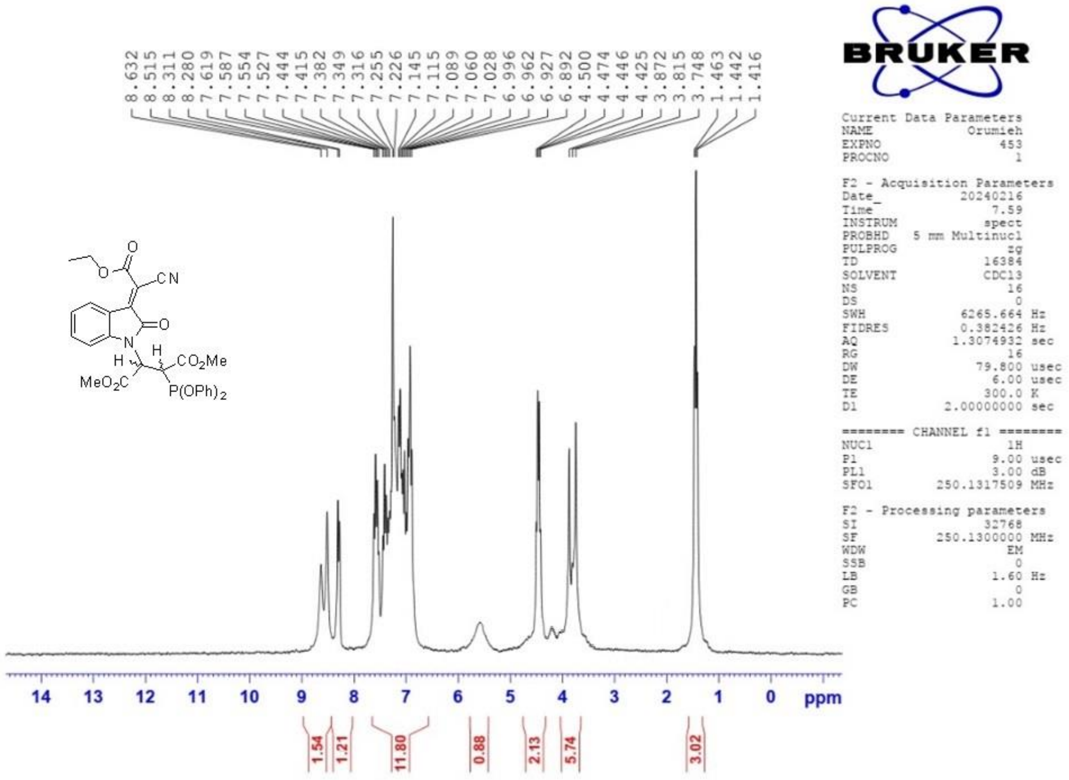
8.632
8.515
8.311
8.280
7.619
7.587
7.554
7.527
7.444
7.415
7.382
7.349
7.316
7.255
7.226
7.145
7.115
7.089
7.060
7.028
6.996
6.962
6.927
6.892
4.500
4.474
4.446
4.425
3.872
3.815
3.748
1.463
1.442
1.416
BRUKER
Current Data Parameters
NAME Orumieh
EXPNO 453
PROCNO 1
F2 - Acquisition Parameters
Date_ 20240216
Time 7.59
INSTRUM spect
PROBHD 5 mm Multinucl
PULPROG zg
TD 16384
SOLVENT CDCl3
NS 16
DS 0
SWH 6265.664 Hz
FIDRES 0.382426 Hz
AQ 1.3074932 sec
RG 16
DW 79.800 usec
DE 6.00 usec
TE 300.0 K
D1 2.00000000 sec
======== CHANNEL f1 ========
NUC1 1H
P1 9.00 usec
PL1 3.00 dB
SFO1 250.1317509 MHz
F2 - Processing parameters
SI 32768
SF 250.1300000 MHz
WDW EM
SSB 0
LB 1.60 Hz
GB 0
PC 1.00
O
CN
O
N
H
H
CO2Me
MeO2C
P(OPh)2
14 13 12 11 10 9 8 7 6 5 4 3 2 1 0 ppm
1.54
1.21
11.80
0.88
2.13
5.74
3.02

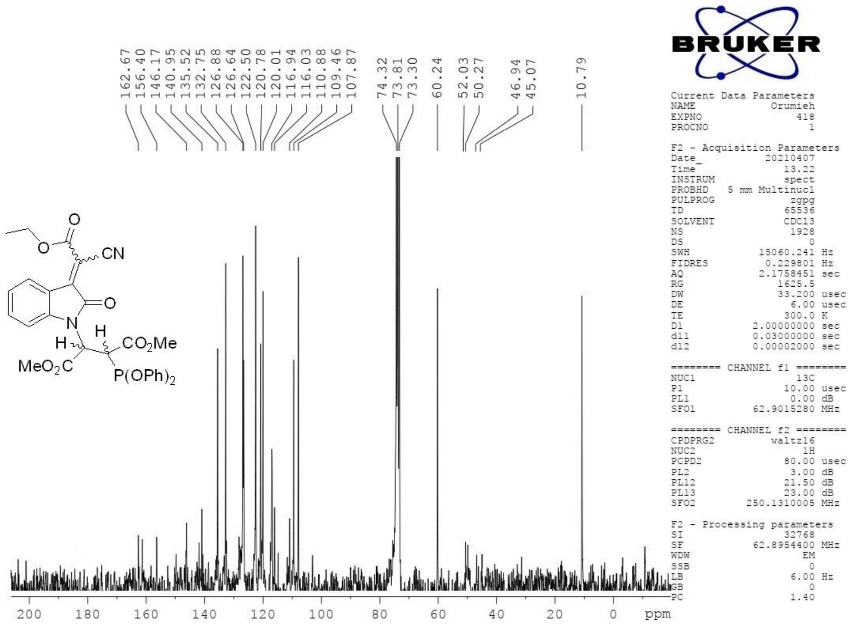
162.67
156.40
146.17
140.95
135.52
132.75
126.88
126.64
122.50
120.78
120.01
116.94
116.03
110.88
109.46
107.87
74.32
73.81
73.30
60.24
52.03
50.27
46.94
45.07
10.79
BRUKER
Current Data Parameters
NAME Orumieh
EXPNO 418
PROCNO 1
F2 - Acquisition Parameters
Date_ 20210407
Time 13.22
INSTRUM spect
PROBHD 5 mm Multinucl
PULPROG zgpg
TD 65536
SOLVENT CDCl3
NS 1928
DS 0
SWH 15060.241 Hz
FIDRES 0.229801 Hz
AQ 2.1758451 sec
RG 1625.5
DW 33.200 usec
DE 6.00 usec
TE 300.0 K
D1 2.00000000 sec
d11 0.03000000 sec
d12 0.00002000 sec
======== CHANNEL f1 ========
NUC1 13C
P1 10.00 usec
PL1 0.00 dB
SFO1 62.9015280 MHz
======== CHANNEL f2 ========
CPDPRG2 waltz16
NUC2 1H
PCPD2 80.00 usec
PL2 3.00 dB
PL12 21.50 dB
PL13 23.00 dB
SFO2 250.1310005 MHz
F2 - Processing parameters
SI 32768
SF 62.8954400 MHz
WDW EM
SSB 0
LB 6.00 Hz
GB 0
PC 1.40
O
CN
O
N
H
H
CO2Me
MeO2C
P(OPh)2
200 180 160 140 120 100 80 60 40 20 0 ppm

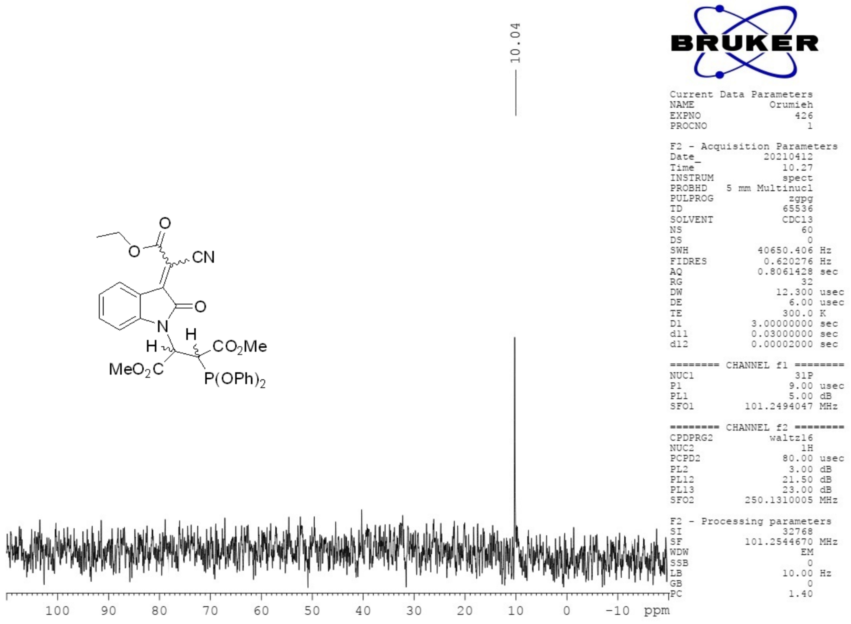
10.04
CN
O
O
O
N
H
H
CO2Me
MeO2C
P(OPh)2
BRUKER
Current Data Parameters
NAME Orumieh
EXPNO 426
PROCNO 1
F2 - Acquisition Parameters
Date_ 20210412
Time 10.27
INSTRUM spect
PROBHD 5 mm Multinucl
PULPROG zgpg
TD 65536
SOLVENT CDCl3
NS 60
DS 0
SWH 40650.406 Hz
FIDRES 0.620276 Hz
AQ 0.8061428 sec
RG 32
DW 12.300 usec
DE 6.00 usec
TE 300.0 K
D1 3.00000000 sec
d11 0.03000000 sec
d12 0.00002000 sec
======== CHANNEL f1 ========
NUC1 31P
P1 9.00 usec
PL1 5.00 dB
SFO1 101.2494047 MHz
======== CHANNEL f2 ========
CPDPRG2 waltz16
NUC2 1H
PCPD2 80.00 usec
PL2 3.00 dB
PL12 21.50 dB
PL13 23.00 dB
SFO2 250.1310005 MHz
F2 - Processing parameters
SI 32768
SF 101.2544670 MHz
WDW EM
SSB 0
LB 10.00 Hz
GB 0
PC 1.40
100 90 80 70 60 50 40 30 20 10 0 -10 ppm
